# Supplementary material for: A Human IPS Model Implicates Embryonic B-Myeloid Fate Restriction as Developmental Susceptibility to B Acute Lymphoblastic Leukemia-Associated ETV6-RUNX1
Source: Dev Cell. 2018 Feb 5;44(3):362–377.e7. doi: 10.1016/j.devcel.2017.12.005 (PMC5807056; doi:10.1016/j.devcel.2017.12.005)
Supplement: Document S1. Figures S1–S5 and Table S1 [file mmc1.pdf]

Developmental Cell, Volume *44*

Supplemental Information

A Human IPS Model Implicates Embryonic B-Myeloid Fate Restriction as Developmental Susceptibility to B Acute Lymphoblastic Leukemia-Associated ETV6-RUNX1

Charlotta Böiers, Simon E. Richardson, Emma Laycock, Alya Zriwil, Virginia A. Turati, John Brown, Jason P. Wray, Dapeng Wang, Chela James, Javier Herrero, Ewa Sitnicka, Stefan Karlsson, Andrew J.H. Smith, Sten Erik W. Jacobsen, and Tariq Enver

SUPPLEMENTAL INFORMATION

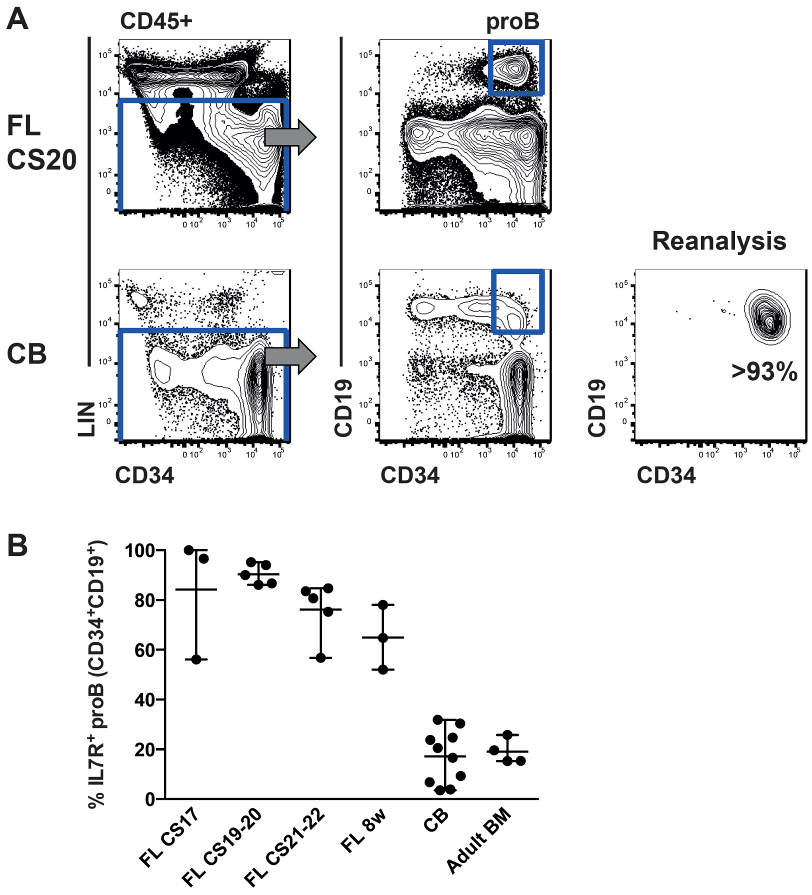

Figure S1 (related to Figure 1)

A. Flow cytometry profile showing gating strategy of proB cells ($CD34^{+}CD19^{+}$). Cells were first gated for viability, singlets and scatter and after that for CD45 positivity. Further gating is indicated in the figure. Reanalysis was normally done on cord blood, due to the low cell number that could be obtained from the fetal samples. ProB cells were typically sorted with >93% purity as indicated in the right panel.
B. Percentage proB cells expressing surface IL7R at different time points of development. Each dot represents one biological sample. Mean with range.

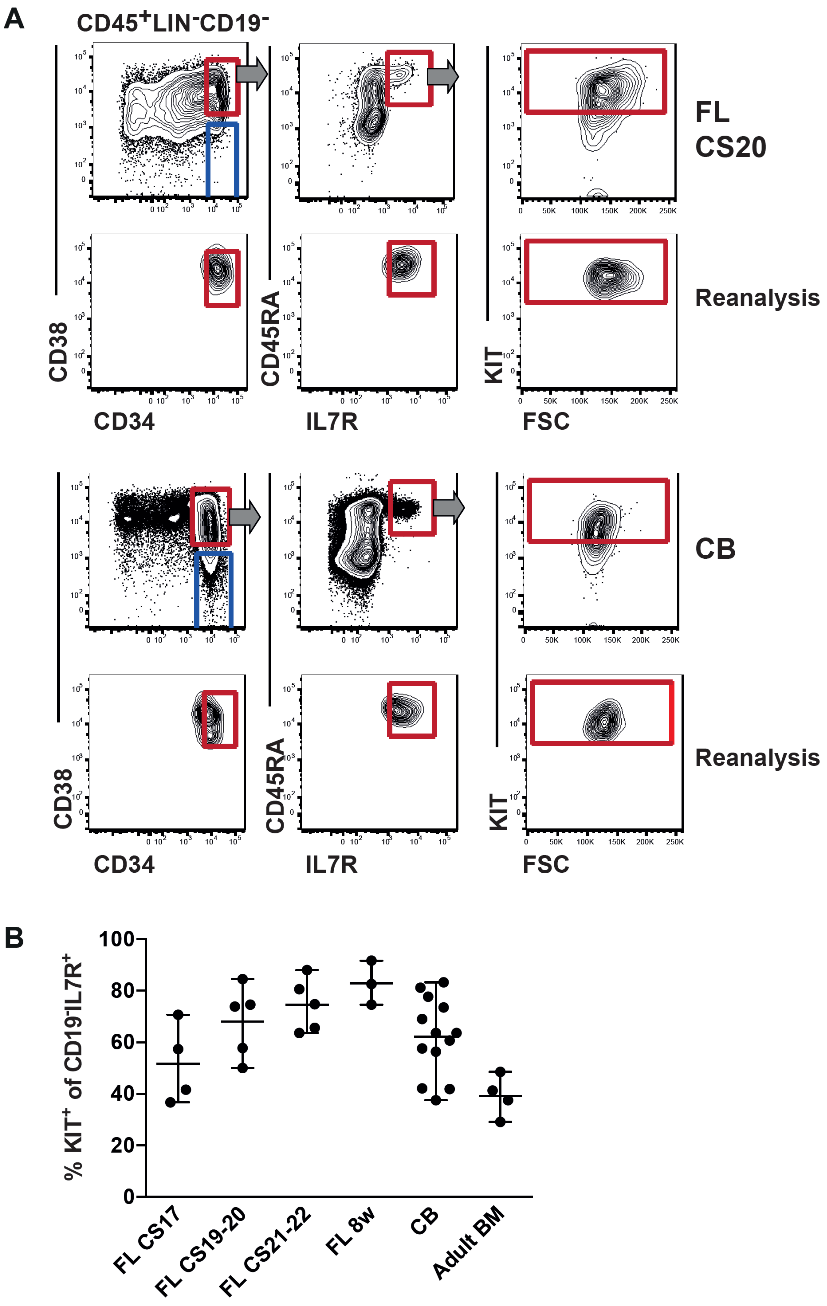

Figure S2 (related to Figure 2)

A. Flow cytometry profiles showing sorting strategy and reanalyses for IL7R$^+$KIT$^+$ progenitors. Viable cells were gated for CD45 positivity, then LIN and CD19 negativity. Further gating as indicated in the figure. CD34$^+$CD38$^-$ gate is indicated in blue. Reanalyses are shown for FL (top) as well as CB (bottom), but were normally done on CB and only occasionally on FL, due to the low cell number that could be obtained from the fetal samples. The IL7R$^+$KIT$^+$ progenitor was typically sorted with >94% purity.
B. Percentage of IL7R$^+$ progenitor cells expressing KIT at different time points of development. Each dot represents one biological sample. Mean with range.

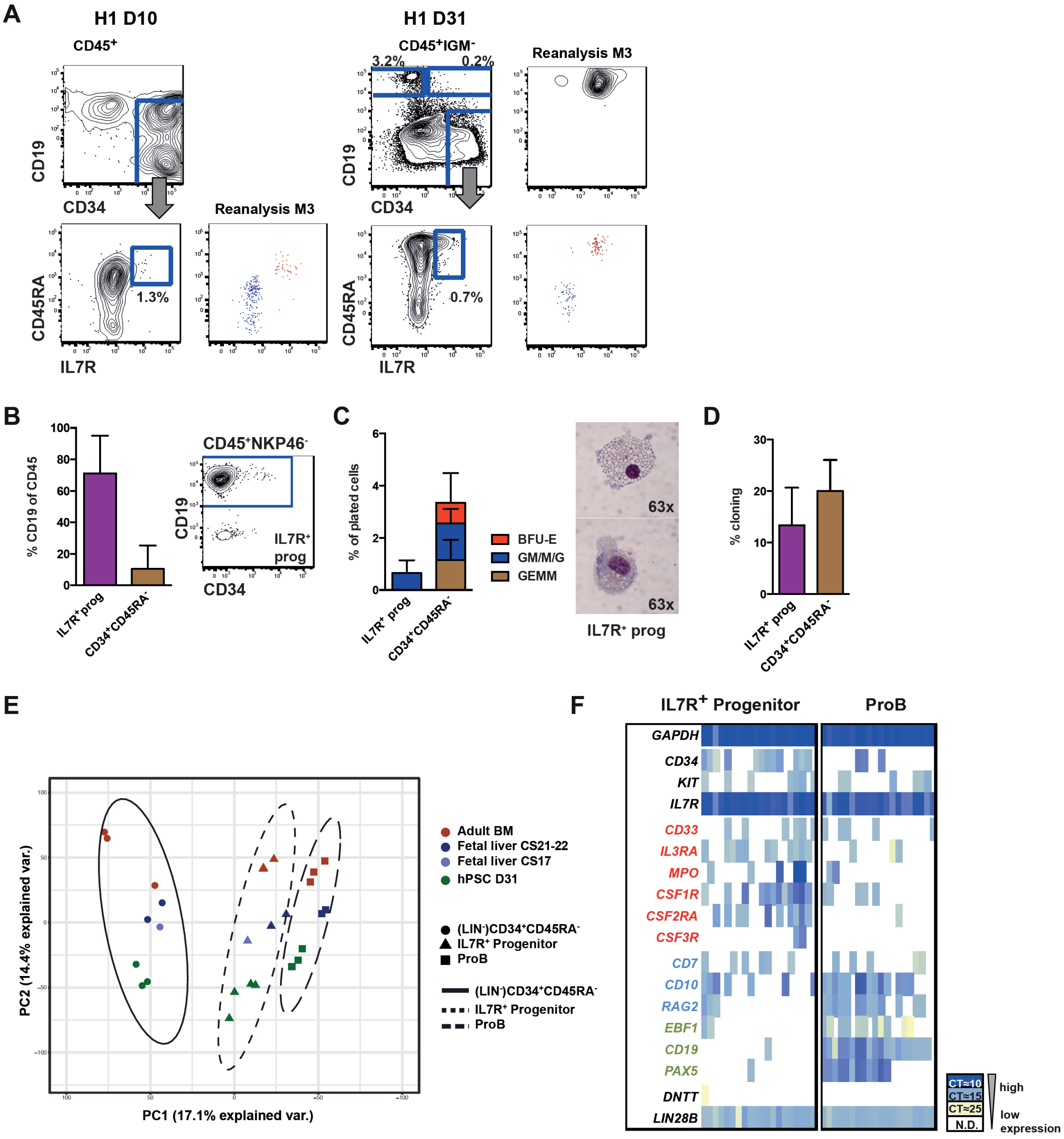

Figure S3 (related to Figure 4)

A. H1 hESCs were analyzed for CD19$^+$ B cells (**top**) and IL7R$^+$ progenitor (**bottom**) at D10 (**left**) and D31 (**right**) of differentiation. Cells were gated CD45$^+$ (D10) or CD45$^+$IGM$^-$ (D31). Further gating is indicated in the figure. Numbers show mean percentages of CD45$^+$ cells. Representative reanalysis of MIFF3 hPSCs is shown to the right of each plot, IL7R$^+$ progenitor (red) and CD34$^+$CD45RA$^-$ control cells (blue), proB cells right top panel. Populations were typically sorted with ≥94% purity.

B. B cell potential of the MIFF3 derived IL7R$^+$ progenitor (D31) was investigated on MS5 stroma. CD34$^+$CD45RA$^-$ cells were cultured in parallel. Graph shows percent CD19$^+$ cells of total CD45$^+$ cells in the culture. Mean+SD, n=2. To the right: representative FACS analysis of the IL7R$^+$ progenitor readout.

C. Myeloid potential of the IL7R$^+$ progenitor (derived from MIFF3 and H1 at D31) was investigated in semi-solid media. Primitive CD34$^+$CD45RA$^-$ cells (also derived from hPSCs) were cultured in parallel. Graph shows percent colonies per plated number of cells. Mean+SD, n=2. To the right: typical macrophages derived from IL7R$^+$ progenitor culture.

D. Myeloid potential of single hPSC-derived IL7R$^+$ progenitors (MIFF3 at D24-27) was investigated in liquid culture. Graph shows percent of wells with >10 cells. Primitive CD34$^+$CD45RA$^-$ cells were cultured in parallel. Mean+SD, n=3.

E. PCA calculated using RNA sequencing data from both human primary samples and hPSCs from D31 ((LIN$^-$)CD34$^+$CD45RA$^-$, IL7R$^+$ progenitor and proB cells) of differentiation. Note that the IL7R progenitor compartment from very early CS17 FL here rests on the correct region of the PCA map whereas it is an outlier on the PCA built on only primary samples (Figure 4D). PC1 and PC2 are shown and each dot represents one individual sample. The ellipses show the 90% density function for that cell type. Legend is shown to the right.

F. Single cell qPCR analysis of IL7R$^+$ progenitor and proB cells from H1 hESCs at D31 of differentiation. Each column represents one single cell. Only cells expressing *GAPDH* are shown. Color code based on CT values. Genes labeled in red are myeloid, blue lymphoid and green B cell genes, n=1, 20 cells investigated/population.

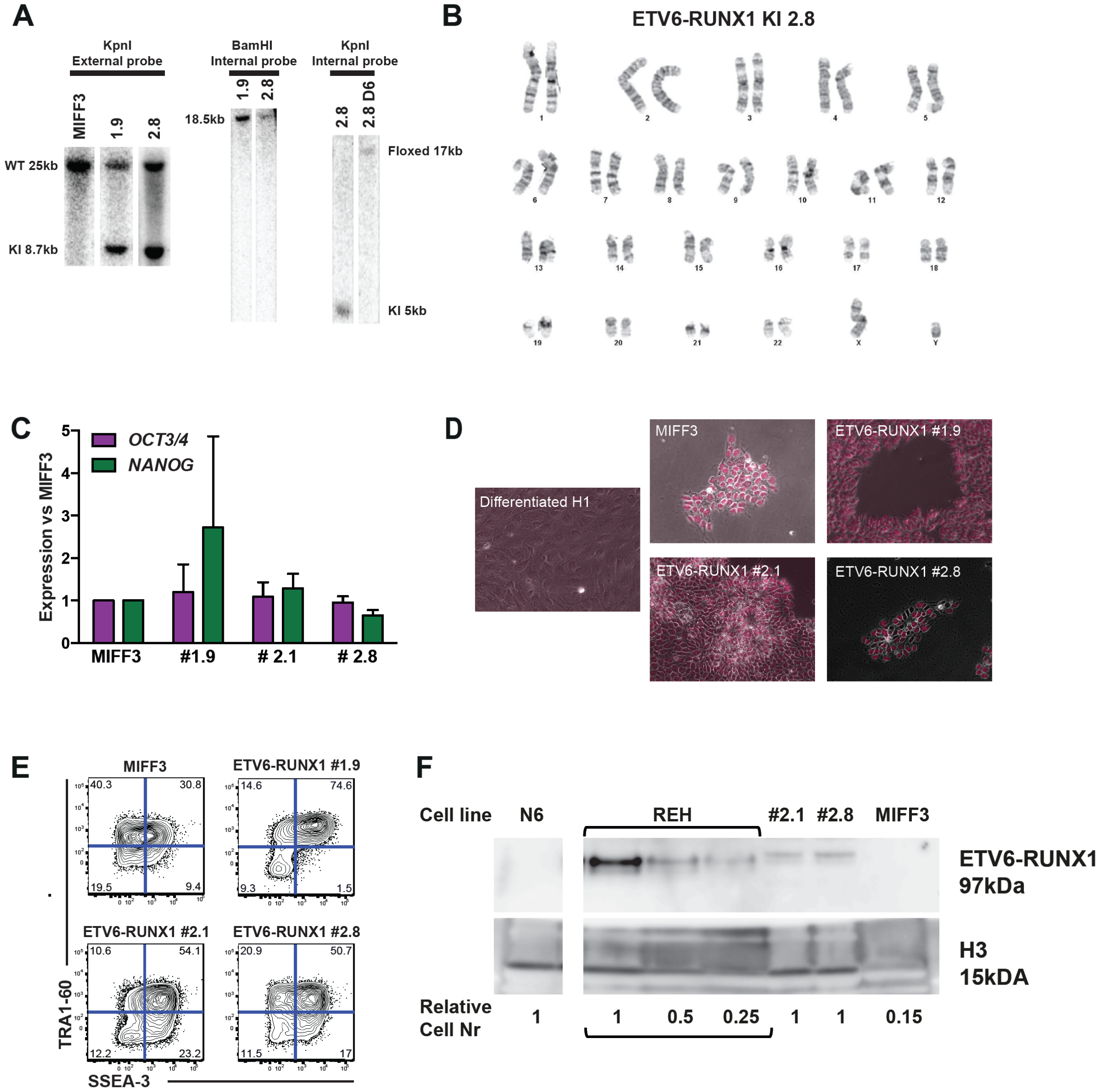

Figure S4 (related to Figure 5)

A. Representative southern blot hybridizations of genome engineered MIFF3 hIPSCs. Left: Successful targeting of *ETV6* allele as shown by external *ETV6* probe on *Kpn*I digested DNA (WT 25kb, KI 8.7kb). Middle: Internal probe (*neoR*) on *Bam*HI digested DNA showing single insertion at 18.5kb. Right: Knock in clone #2.8 before (left - KI 5kb) and after (right - reverted 17kb) floxing with cre recombinase using internal (*neoR*) probe on *KpnI* digested DNA.
B. Karyotype of middle passage ETV6-RUNX1 knock-in hPSC line #2.8.
C. Quantitative gene-expression analysis of unmodified MIFF3 and 3 successfully targeted ETV6-RUNX1 knock in clones for the pluripotency genes *OCT3/4* and *NANOG*. Expression is normalized to *β ACTIN* and presented relative to MIFF3. Mean+SD, n=2.
D. Immunostaining for nuclear OCT4 on fixed MIFF3 and 3 different ETV6-RUNX1 knock-in clones. Differentiated H1 is shown as control.

E. Flow cytometry analysis of the pluripotency surface markers TRA1-60 and SSEA3 in MIFF3 and 3 different ETV6-RUNX1 knock-in clones.
F. Western blot of ETV6-RUNX1 protein from FACS sorted hematopoietic progenitors ($CD45^{+}Venus^{+}$) derived from ETV6-RUNX1 knock in #2.1 and #2.8. Positive control: ETV6-RUNX1 B-ALL cell line (REH). Negative controls: MIFF3 and NALM6 (N6). Note knock-in ETV6-RUNX1 protein includes additional V5 tag. One lane has been removed but all samples were run on the same gel.

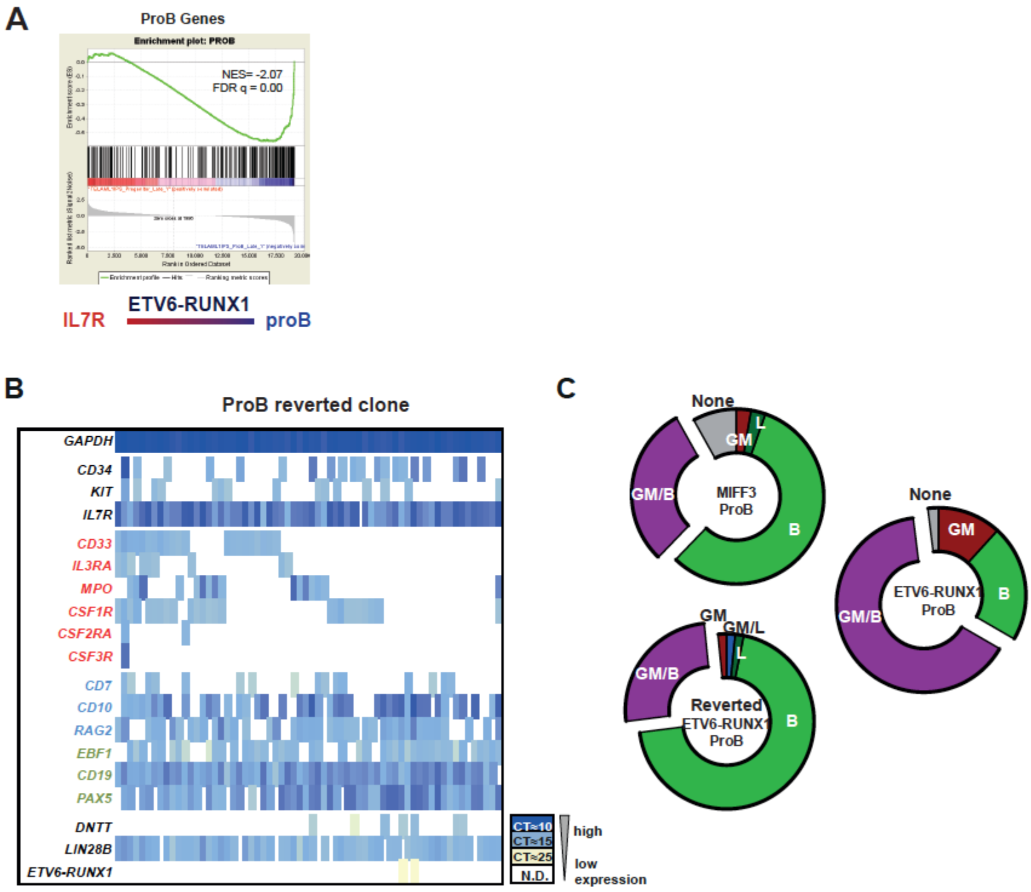

Figure S5 (related to Figure 6)

A. Gene set enrichment analysis (GSEA) comparing the proB cell gene expression of ETV6-RUNX1^{+} IL7R progenitor and ETV6-RUNX1^{+}proB cells.
B. Single cell qPCR analysis of proB cells (CD34^{+}CD19^{+}) from ETV6-RUNX1 reverted clone. Each column represents one single cell. Only cells expressing *GAPDH* are shown. Color code according to CT values. Gene sets: red, myeloid; blue, lymphoid; green, B lineage related. n=2, 64 cells investigated.
C. Co-expression of lineage associated genes based on single cell data in Figure 6E, and Figure S5B. A cell is considered myeloid (GM) if expressing 2 of the myeloid genes, lymphoid (L) if 2 of the genes listed in blue and B primed (B) if expressing 2 of the genes listed in green. Co-expression of GM with L or B genes are labeled GM/L and GM/B respectively (see *STAR Method*). Data from D31 proB are shown (MIFF3, ETV6-RUNX1 hIPS and ETV6-RUNX1 hIPS reverted clones).

***Table S1:* (related to STAR Methods)**

Oligonucleotides

Oligonucleotides	Source	Identifier	Used for PCA
CCR9	ThermoFisher	Hs00258535_m1	x
CD10	ThermoFisher	Hs00153510_m1	x
CD19	ThermoFisher	Hs00174333_m1	x
CD33	ThermoFisher	Hs01076281_m1	x
CD34	ThermoFisher	Hs00990732_m1	x
CD38	ThermoFisher	Hs01120071_m1	x
CD62L (SELL)	ThermoFisher	Hs00174151_m1	x
CD7	ThermoFisher	Hs00196191_m1	x
CKIT	ThermoFisher	Hs00174029_m1	x
CRLF2	ThermoFisher	Hs00845692_m1	x
CSF1R (M-CSFR)	ThermoFisher	Hs00911250_m1	x
CSF2RA (GM-CSFR)	ThermoFisher	Hs00538900_m1	x
CSF3R (G-CSF R)	ThermoFisher	Hs01114427_m1	x
DNTT	ThermoFisher	Hs00172743_m1	x
E2A (TCF-3)	ThermoFisher	Hs00413032_m1	x
EBF1	ThermoFisher	Hs00395513_m1	x
EPOR	ThermoFisher	Hs00181092_m1	x
ETV6	ThermoFisher	HS00231101_m1	
*ETV6-RUNX1**	ThermoFisher	F *TEL*: CTCTGTCTCCCCGCCTGAA<br>P *TEL*; TCCCAATGGGCATGGCGTGC<br>R *AML1*; CGGCTCGTGCTGGCA	
FLT3	ThermoFisher	Hs00174690_m1	x
GATA1	ThermoFisher	Hs01085823_m1	x
GATA3	ThermoFisher	Hs00231122_m1	x
GAPDH	ThermoFisher	Hs99999905_m1	x
IGHM	ThermoFisher	Hs00378435_m1	x
IKAROS	ThermoFisher	Hs00172991_m1	x
IL2RG	ThermoFisher	Hs00953624_m1	x
IL3RA	ThermoFisher	Hs00608141_m1	x
IL7R	ThermoFisher	Hs00902334_m1	x
LIN28B	ThermoFisher	Hs01013729_m1	x
MPL	ThermoFisher	Hs00180489_m1	x
MPO	ThermoFisher	Hs00165162_m1	x
NOTCH1	ThermoFisher	Hs01062011_m1	x
PAX5	ThermoFisher	Hs00277134_m1	x
PRETa	ThermoFisher	Hs00300125_m1	x
PU.1	ThermoFisher	Hs02786711_m1	x
RAG2	ThermoFisher	Hs00379177_m1	x
TCF7	ThermoFisher	Hs00175273_m1	x
VWF	ThermoFisher	Hs00169795_m1	x
OCT3/4	IDT	F: GAAGGAGAAGCTGGAGCAAA	N/A

		R: CTTCTGCTTCAGGAGCTTG	N/A
NANOG	IDT	F: GATTTGTGGGCCTGAAGAAA<br>R: CAGGGCTGTCCTGAATAAGC	N/A<br>N/A
β-ACTIN	ThermoFisher	Hs99999903_m1	N/A
D_H1	IDT/Sigma	GGCGGAATGTGTGCAGGC	N/A
D_H2	IDT/Sigma	GCACTGGGCTCAGAGTCCTCT	N/A
D_H3	IDT/Sigma	GTGGCCCTGGGAATATAAAA	N/A
D_H4	IDT/Sigma	AGATCCCCAGGACGCAGCA	N/A
D_H5	IDT/Sigma	CAGGGGGACACTGTGCATGT	N/A
D_H6	IDT/Sigma	TGACCCCAGCAAGGGAAGG	N/A
D_H7	IDT/Sigma	CACAGGCCCCCTACCAGC	N/A
JH consensus	IDT/Sigma	CTTACCTGAGGAGACGGTGACC	N/A
5' gap-repair<br>3' gap-repair	IDT<br>IDT	GCTAATATATATAATATATTATATATACGAATATACTAATTATGGCTGACTTAATA AGATGATCTTCTTGAGATCG<br>CGTAAAAGTTCTGTCATTAACTGAATAGCCATAAAAAGCCTTTAAATCTCTTACC AATGCTTAATCAGTGAGG	
5' rpsL-genta<br>3' rpsL-genta	IDT<br>IDT	CACCATTCCCAATTAGGCGCCCTCCAAGGCTCTCTGAGGGCAATTGGAGGCATTA ATTCCTAATTTTTGTTGAC<br>AGCGGCTCATTAACCAAGCCATTAGGGATTTAGCCTCATCCAAGCAGAAGTTAGG TGGCGGTACTTGGG	
ETV6 Southern probe F<br>*ETV6* Southern probe R	IDT<br>IDT	CCCATCTGAGAGGGACTGTG<br>GTATCCGCAGCTAAAGGCATTG	N/A<br>N/A
neoR Southern probe F<br>*neoR* Southern probe R	IDT<br>IDT	GCTATTCGGCTATGACTGGG<br>GAAGGCGATAGAAGGCGATG	N/A<br>N/A

* Gabert et al., *Leukemia*, 2003
