# Supplementary material for: A Human IPS Model Implicates Embryonic B-Myeloid Fate Restriction as Developmental Susceptibility to B Acute Lymphoblastic Leukemia-Associated ETV6-RUNX1
Source: Dev Cell. 2018 Feb 5;44(3):362–377.e7. doi: 10.1016/j.devcel.2017.12.005 (PMC5807056; doi:10.1016/j.devcel.2017.12.005)
Supplement: Document S2. Article plus Supplemental Information [file mmc2.pdf]

Article

Developmental Cell

A Human IPS Model Implicates Embryonic B-Myeloid Fate Restriction as Developmental Susceptibility to B Acute Lymphoblastic Leukemia-Associated ETV6-RUNX1

Highlights

- High-resolution characterization of the earliest human B lineage progenitors
- Fetal CD19^{-}IL-7R^{+} progenitors uniquely co-express B/myeloid genes during development
- Lymphoid commitment in hPSC culture recapitulates human fetal B lymphopoiesis
- hPSC-derived proB cells expressing ETV6-RUNX1 aberrantly co-express myeloid genes

Authors

Charlotta Böiers, Simon E. Richardson, Emma Laycock, ..., Andrew J.H. Smith, Sten Erik W. Jacobsen, Tariq Enver

Correspondence

t.enver@ucl.ac.uk

In Brief

Böiers, Richardson et al. explore the potential for a developmental susceptibility to childhood acute lymphoblastic leukemia. Characterization of earliest B cell progenitors in human fetal liver identified a unique progenitor compartment that can be recapitulated using human pluripotent stem cells to model the impact of the pre-leukemia-initiating oncogene ETV6-RUNX1.

Böiers et al., 2018, Developmental Cell *44*, 362–377
February 5, 2018 © 2017 The Author(s). Published by Elsevier Inc.
https://doi.org/10.1016/j.devcel.2017.12.005

Developmental Cell
Article

A Human IPS Model Implicates Embryonic B-Myeloid Fate Restriction as Developmental Susceptibility to B Acute Lymphoblastic Leukemia-Associated ETV6-RUNX1

Charlotta Böiers,[1,2,8] Simon E. Richardson,[1,8] Emma Laycock,[1] Alya Zriwil,[2] Virginia A. Turati,[1] John Brown,[1] Jason P. Wray,[1] Dapeng Wang,[1] Chela James,[1] Javier Herrero,[1] Ewa Sitnicka,[2] Stefan Karlsson,[2] Andrew J.H. Smith,[3,4] Sten Erik W. Jacobsen,[4,5,6,7] and Tariq Enver[1,2,9,*]
[1]Department of Cancer Biology, UCL Cancer Institute, UCL, London, UK
[2]Lund Stem Cell Center, Lund University, Lund, Sweden
[3]MRC Centre for Regenerative Medicine, University of Edinburgh, Edinburgh, UK
[4]MRC Molecular Haematology Unit, University of Oxford, Oxford, UK
[5]Departments of Cell and Molecular Biology and Medicine Huddinge, Center for Hematology and Regenerative Medicine, Karolinska Institutet, Stockholm, Sweden
[6]Haematopoietic Stem Cell Laboratory, Weatherall Institute of Molecular Medicine, University of Oxford, Oxford, UK
[7]Karolinska University Hospital, Stockholm, Sweden
[8]These authors contributed equally
[9]Lead Contact
*Correspondence: t.enver@ucl.ac.uk
https://doi.org/10.1016/j.devcel.2017.12.005

SUMMARY

ETV6-RUNX1 is associated with childhood acute B-lymphoblastic leukemia (cALL) functioning as a first-hit mutation that initiates a clinically silent pre-leukemia *in utero*. Because lineage commitment hierarchies differ between embryo and adult, and the impact of oncogenes is cell-context dependent, we hypothesized that the childhood affiliation of ETV6-RUNX1 cALL reflects its origins in a progenitor unique to embryonic life. We characterize the first emerging B cells in first-trimester human embryos, identifying a developmentally restricted CD19$^-$IL-7R$^+$ progenitor compartment, which transitions from a myeloid to lymphoid program during ontogeny. This developmental series is recapitulated in differentiating human pluripotent stem cells (hPSCs), thereby providing a model for the initiation of cALL. Genome-engineered hPSCs expressing ETV6-RUNX1 from the endogenous *ETV6* locus show expansion of the CD19$^-$IL-7R$^+$ compartment, show a partial block in B lineage commitment, and produce proB cells with aberrant myeloid gene expression signatures and potential: features (collectively) consistent with a pre-leukemic state.

INTRODUCTION

Childhood acute lymphoblastic leukemia (cALL) is clinically distinct from that in adults, with higher incidence, better prognosis, and a distinct mutational spectrum. Analysis of neonatal blood spots and monochorionic twins of ALL cases have demonstrated that the initiating mutations are frequently acquired *in utero* (Greaves and Wiemels, 2003). One possibility for this is that fetal B lineage progenitors have a developmental susceptibility to pre-leukemic initiation. We therefore hypothesized that the distinct features of childhood ALL are due in part to its initiation in a transient progenitor compartment with B lineage potential unique to early human development.

ETV6-RUNX1 (TEL-AML1) accounts for 25% of precursor B cell ALL (B-ALL) in children, but is seldom seen in adult ALL. Evidence from both neonatal blood spots and monochorionic twins of ETV6-RUNX1 ALL cases, supported by deep sequencing, has demonstrated that ETV6-RUNX1 frequently arises *in utero*, is unequivocally an initiating event (Greaves, 2003; Papaemmanuil et al., 2014), and occurs at high frequency, with approximately 1% of neonates harboring this first-hit translocation (Mori et al., 2002). However, only 1% of children with the ETV6-RUNX1 mutation develop the necessary second-hit mutations to transform to overt ALL, indicating that this is a common, but weakly penetrant first-hit oncogene. These secondary mutations are acquired in a branching polytypic evolutionary pattern (Anderson et al., 2011), although the exact mechanisms underlying transformation to ALL remain uncertain. Deep-sequencing studies have shown most second-hit mutations have evidence of recombination activating gene (RAG)-mediated mutagenesis, while other studies have implicated activation-induced cytidine deaminase-mediated mutagenesis, exposure to infective agents, and aberrant response of B cell precursors to cytokine signaling (Ford et al., 2009; Greaves, 2006; Papaemmanuil et al., 2014; Swaminathan et al., 2015).

Understanding the natural history of pre-leukemic initiation and subsequent transformation requires knowledge of both the

 © 2017 The Author(s). Published by Elsevier Inc.
This is an open access article under the CC BY license (http://creativecommons.org/licenses/by/4.0/).

cell in which the first-hit mutation arises, but most pertinently the downstream compartments that are impacted. Single-cell *IGH* and *TCR* sequencing of monochorionic twins with ETV6-RUNX1 childhood ALL have been particularly informative, identifying common ancestral clones containing partial IGH_{DJ} or even *TCR* rearrangements (Alpar et al., 2015). This strongly indicates that leukemic transformation occurs within an early *RAG*-expressing multipotent lymphoid progenitor, which has either been trapped by differentiation arrest and/or expanded by ETV6-RUNX1. In addition, the failure to identify polyclonal IgH DJ or TCR rearrangements in ETV6-RUNX1 pre-leukemia make it unlikely that the ETV6-RUNX1 pre-leukemic hierarchy is maintained by a more primitive RAG^{-} hematopoietic stem cell (HSC)-like population (Alpar et al., 2015; Hong et al., 2008). This notwithstanding, the clinical features of aberrant co-expression of myeloid antigens and lineage switching commonly seen in many childhood B-ALLs indicate that the disease may arise in a target progenitor cell that normally harbors some degree of myeloid as well as lymphoid lineage programming (Gerr et al., 2010).

Detailed studies of mouse embryonic hematopoiesis have characterized developmental differences with multiple sites of specification, niches, and the production of functionally distinct effector cells (Medvinsky et al., 2011; Yuan et al., 2012). Blood, mainly erythrocytes and macrophages, first arises in the yolk sac (Tavian and Peault, 2005), whereas the first definitive HSCs (dHSCs) are formed in the aorta gonad mesonephros (AGM) region in both mouse (at embryonic day 10.5) and human (around 30 days post fertilization) (Ivanovs et al., 2011; Medvinsky and Dzierzak, 1996). HSCs and progenitors migrate from the AGM to the fetal liver (FL), which becomes the main site of hematopoiesis before the bone marrow (BM) takes over just before birth (Tavian and Peault, 2005).

Development of the adaptive immune system (B and T cells) was believed to occur after the emergence of dHSCs at the AGM; however, a recent study detected murine fetal interleukin-7 receptor ($IL\text{-}7R^{+}$) *Rag1*-expressing lymphoid progenitor much earlier (Boiers et al., 2013). This early immune-restricted progenitor co-expressed myeloid and lymphoid lineage programs and contributed to both myeloid and lymphoid lineages in the embryo, whereas in the adult *Rag-1* progenitors contributed almost exclusively to the lymphoid lineage. This murine study indicates important differences in the lineage specification of fetal and adult B cell progenitors, which, if found in the human, might underlie a developmental susceptibility to pre-leukemic initiation by fusion transcription factors such as ETV6-RUNX1.

There have been several attempts to model pre-leukemic initiation by ETV6-RUNX1 in mouse and human. These have produced variable results as to the molecular mechanism of ETV6-RUNX1 and have implicated a target cell anywhere from HSC to B lineage-restricted cells (Hong et al., 2008; Schindler et al., 2009; van der Weyden et al., 2011). Furthermore, ETV6-RUNX1 expression level has been shown to affect the observed phenotype, raising concerns over the veracity of models using viral transgenesis (Tsuzuki and Seto, 2013). Of note, authentic B lineage ALL in response to ETV6-RUNX1, with or without the second hits found in ETV6-RUNX1 patients, has not been reliably seen in non-human model systems (van der Weyden et al., 2011). Together this implies that ETV6-RUNX1 exerts a relatively subtle first-hit activity, and that any model of the pre-leukemic effect of ETV6-RUNX1 requires a developmentally relevant human system expressing physiological levels of ETV6-RUNX1.

We hypothesized that the distinct features of childhood ALL are due in part to its initiation in a transient progenitor compartment with B lineage potential unique to early human development. Thus, to establish the authentic first-hit impact of the cALL oncogene ETV6-RUNX1 necessitates its expression in the transcriptional context of the appropriate developmental stage. Studies of both mouse and human embryonic hematopoiesis have demonstrated unique progenitor states during development (Boiers et al., 2013; Notta et al., 2015), and it is increasingly understood that oncogenic mutations can have distinct effects on cell fate in different developmental contexts (Horton et al., 2013; Man et al., 2016; Porter et al., 2016). Understanding the interaction of leukemia-initiating mutations with developmentally restricted cell states requires a model of the relevant stages of human fetal B lymphopoiesis. While this is almost impossible using primary material from human fetuses, *in vitro* differentiation of human pluripotent stem cells (hPSCs) potentially offers a tractable system to model early embryonic hematopoiesis (Slukvin, 2013), although it remains unclear which developmental hematopoietic hierarchy it recapitulates. hPSCs are known to produce cells expressing embryonic hemoglobins, and attempts to produce transplantable dHSCs from hPSCs have been inconsistent (Slukvin, 2013). If hPSC-derived B cell precursors recapitulate important developmental characteristics of the earliest B lymphoid progenitor cells in the human embryo, then hPSCs could provide a tractable model to explore the impact of cALL oncogenes on this currently inaccessible arena of human development.

We have characterized B lymphoid development in first-trimester human embryos, identifying an $IL\text{-}7R^{+}$ progenitor compartment that transitions from myeloid to lymphoid programming during development, resulting in a transient population that co-expresses myeloid and B lymphoid genes. We demonstrate that hPSCs recapitulate this fetally distinct B cell progenitor hierarchy, providing a developmentally relevant model of early embryonic B lymphopoiesis. ETV6-RUNX1 expressed at physiological levels from the *ETV6* promoter in genome-engineered hPSCs specifically affects the transition from fetal $IL\text{-}7R^{+}$ progenitor compartment to committed proB cell. We therefore propose that the lineage dynamics of the fetal $IL\text{-}7R^{+}$ compartment are particularly susceptible to dysregulation by ETV6-RUNX1, thereby providing an explanation for the childhood restriction of cALL.

RESULTS

The Majority of Emerging B Cells in the Human FL Express IL-7R

We examined first-trimester human FL aiming to identify the earliest emerging $CD19^{+}$ B cells and compare them with neonatal cord blood (CB) and adult BM B cells (schematic overview of ontogeny; Figure 1A). At Carnegie stage (CS) 17 we found a small population of $CD19^{+}$ B cells in three out of four FL samples (<0.5% of total $CD45^{+}$ blood cells). These $CD19^{+}$ B cells co-expressed the progenitor marker CD34, consistent with the proB stage of development (Figures 1B and 1C, top panel) (Hystad

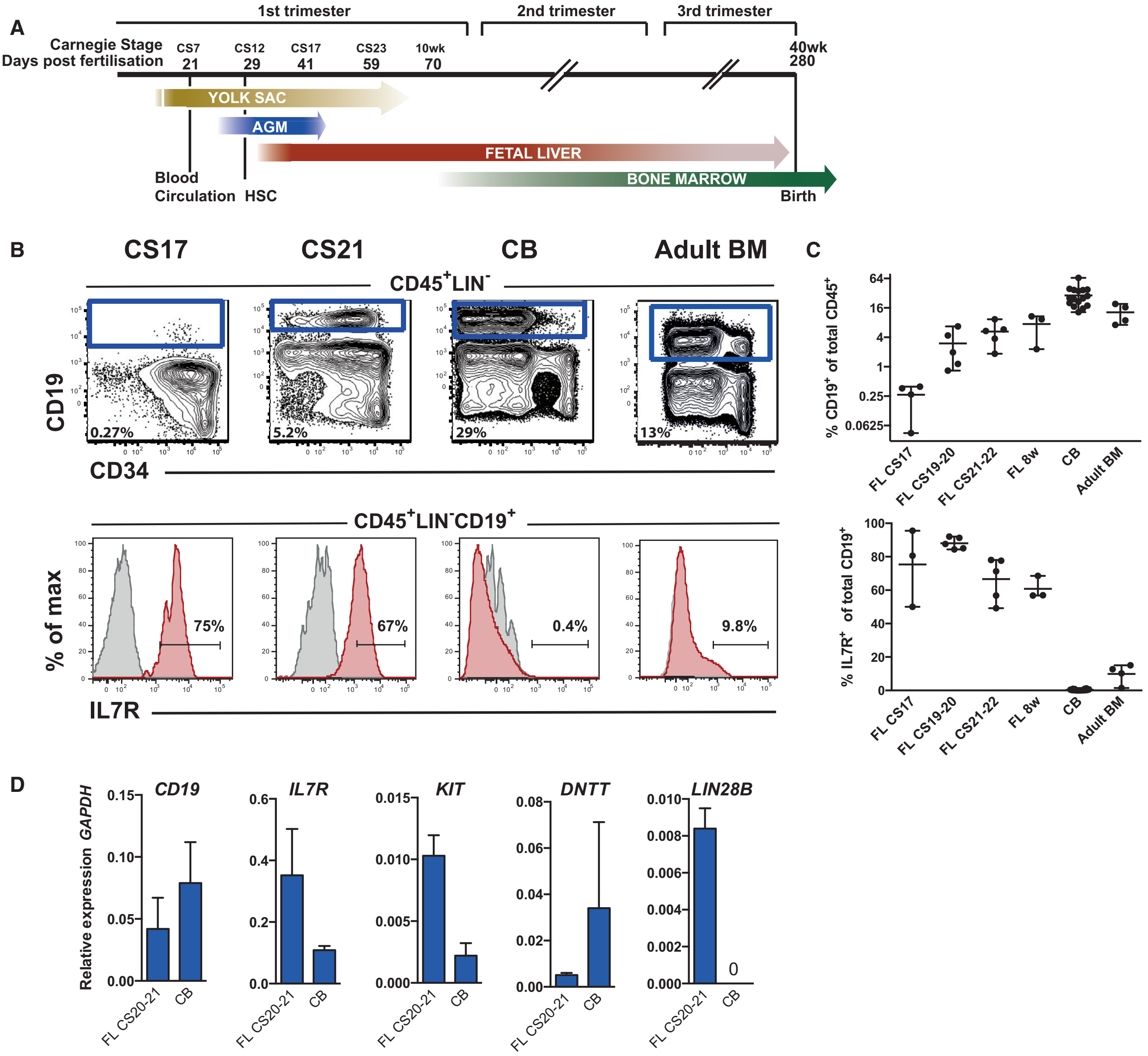

Figure 1. The Majority of Emerging B Cells in the Human Fetal Liver Express IL-7R

(A) Major hematopoietic sites in the developing human embryo. Timescale shows days of gestation and embryonic development by Carnegie stage (CS) (Tavian and Peault, 2005).

(B) CD19$^+$ B cells from human fetal livers (FLs) at CS17 and CS21-22, cord blood (CB) and adult bone marrow (BM) were analyzed for surface expression of IL-7R. Viable cells were gated CD45$^+$LIN$^-$, further gating as indicated. Top panel: mean percentage CD19$^+$ B cells of total CD45$^+$ cells. Bottom panel: mean percentage of CD19$^+$ B cells expressing IL-7R.

(C) Emergence of CD19$^+$ B cells as percent of total CD45$^+$ cells (top panel) and percent CD19$^+$ B cells expressing IL-7R (bottom panel) at different time points of development. Each dot represents one biological sample. Mean with range.

(D) Quantitative gene-expression analysis of CD34$^+$CD19$^+$ proB cells (CS 20–21 FL and CB). Data are presented relative to *GAPDH*; mean ± SD, n = 2–3. See also Figure S1.

et al., 2007). By CS20 we saw the emergence of more mature CD34$^-$CD19$^+$ preB cells (Figures 1B, 1C, and S1A). We looked for surface markers that could discriminate between fetal and adult B cells. The majority of FL CD19$^+$ B cells expressed IL-7R, with as many as 60%–88% FL CD19$^+$ B cells being IL-7R$^+$, whereas in adult BM a much smaller fraction were IL-7R$^+$ (9.8% total CD19$^+$ and 19% proB) (Figures 1B and 1C, bottom panel, and S1B). This was confirmed by qPCR (Figure 1D, sorting profiles Figure S1A). Expression of the tyrosine kinase receptor *KIT*, also implicated in regulation of B lymphopoiesis (Waskow et al., 2002), was higher in the FL proB cells, which we confirmed by flow cytometry (data not shown), whereas *DNTT* (encoding

TDT) was higher in neonatal CB proB cells, consistent with studies in mice (Li et al., 1993). Embryonic hematopoietic tissues are known to express *LIN28B* and we found it to be upregulated in FL proB cells compared with CB (Yuan et al., 2012).

Identification of a CD19^{-}IL-7R^{+} Progenitor

We next looked for a candidate upstream progenitor of the fetal proB cells identified above. As surface expression of IL-7R was so prominent in emerging proB cells, we hypothesized that they may arise from a more primitive CD19^{-}IL-7R^{+} progenitor. We explored hematopoietic progenitor compartments (CD45^{+}CD34^{+}), excluding cells expressing lineage commitment markers (LIN: CD19, CD3, CD8a, CD56, and CD235a), and identified a candidate LIN^{-}IL-7R^{+} population in the early FL. In common with fetal proB cells the IL-7R^{+} population also expressed KIT (CD117), CD45RA, and the activation marker CD38 (Figures 2A, S2A, and S2B). The frequency of cells with this immunophenotype as a proportion of the CD45^{+}CD34^{+} progenitor compartment decreased markedly through development with a 7- to 10-fold difference between CS17 FL and CB or adult BM (Figure 2B).

We tested the capacity of the IL-7R^{+} population to form B cells by co-culture on MS-5 stroma. The FL CD19^{-}IL-7R^{+} cells (LIN^{-}CD19^{-}CD34^{+}CD38^{+}CD45RA^{+}IL-7R^{+}KIT^{+}) efficiently produced CD19^{+} B cells (Figure 2C, sort profiles Figure S2A) demonstrating that this population is indeed a B cell progenitor (henceforth referred to as the IL-7R progenitor). That the IL-7R progenitor robustly formed B cells *in vitro* prompted us to investigate if the progenitor had initiated rearrangement of the immunoglobulin heavy chain (van Dongen et al., 2003). We found that the IL-7R progenitor had evidence of DJ_H rearrangement (Figure 2D), although, as expected, fewer cells appeared DJ rearranged than in the proB cell population.

The fetal IL-7R progenitor compartment was also capable of producing myeloid output of predominantly macrophage lineage in both semi-solid and liquid culture, with a frequency equivalent to that from a CD34^{+} primitive control fraction (Figures 2E and 2F). No detectable erythroid potential was seen in semi-solid culture, indicating that the IL-7R progenitor is immune restricted (Figure 2E).

The IL-7R Progenitor Transitions from a Myeloid to a Lymphoid Gene Expression Signature during Early Embryonic Development

Consistent with the lymphoid and myelomonocytic outputs seen from this population *in vitro*, we observed evidence of both myeloid- and lymphoid-affiliated gene expression by qPCR analysis of bulk IL-7R progenitor cells (data not shown). To understand whether these programs were co-expressed within single cells, we performed single-cell expression analysis of lineage-associated genes at different stages of development (Figure 3A).

As expected, the FL IL-7R progenitor expressed the fetal marker *LIN28B*, which was not seen in adult BM; conversely, the adult BM IL-7R progenitor expressed *DNTT*, which was not seen in the FL (Li et al., 1993; Yuan et al., 2012). Overall, the earliest IL-7R progenitor cells from CS17 expressed predominantly myeloid genes, with the majority of cells lacking an identifiable or coherent lymphoid program. By CS20, early lymphoid- and B cell-specific programs were expressed while retaining a myeloid signature, suggesting that the IL-7R progenitor had transitioned from a myeloid-primed to a lympho-myeloid-primed transcriptional state (Figure 3A). Consistent with the identification of DJ rearrangements in the *IgH* gene from the FL IL-7R progenitor (Figure 2D), *RAG2* is expressed in almost all of the IL-7R^{+} progenitor cells we tested from this stage of development. Collectively, these transcriptional data are consistent with the functional data showing myeloid and lymphoid output from the FL IL-7R progenitor at the population level (Figures 2C, 2E, and 2F). To confirm that any given cell within this population truly had dual lympho-myeloid potential, we assessed *IGH* DJ status within myeloid cells derived from this population. The presence of DJ rearrangements within these myeloid cells demonstrates their origin in an RAG-expressing lymphoid progenitor (Figure 3B).

We next evaluated the extent to which lymphoid and myeloid programs were co-resident in individual cells through development. Cells were operationally considered myeloid primed if expressing two or more of *CD33*, *IL3RA*, *MPO*, *CSF1R*, *CSF2RA*, and *CSF3R*, lymphoid primed if expressing two or more of *CD7*, *CD10*, and *RAG2*, or B committed if expressing two or more of *EBF1*, *PAX5*, and *CD19*. This analysis revealed a transition from a primarily myeloid progenitor state at CS17, to a predominantly lymphoid state in CB and adult BM, transiting through a B-myeloid (GM/B) state at CS20, which was rare or not present at other (CB and adult BM) developmental stages (Figure 3C). Qualitatively, the lymphoid program appeared different in adult BM from CS20 FL, with higher expression of a core lymphoid program, but less consistent expression of B lineage-restricted genes.

To determine whether the IL-7R progenitor and proB cells from different stages of development could be objectively separated by their overall pattern of gene expression, we analyzed the single-cell qPCR data by principal-component analysis (PCA) (Figure 3D). The proB cell clusters from FL, CB, and adult BM overlapped, with proB cells from adult BM displaying the least heterogeneity. However, the IL-7R progenitor compartments from different developmental stages formed distinct clusters along a developmental axis, with the early CS17 FL progenitor also segregating from later CS20 FL IL-7R progenitors. Genes contributing most strongly to the signature of the early FL IL-7R progenitor included the myeloid tyrosine kinase receptor *CSF1R* (M-CSFR) and *LIN28B*.

Lymphoid Commitment in hPSC Culture Recapitulates Human Fetal B Lymphopoiesis

We speculated that hPSCs might be able to model the characteristics of fetal lymphopoiesis. For these studies we used a human induced PSC (hIPSC) line, MIFF3, and corroborated our findings using the H1 human embryonic stem cell (hESC) line. An OP9/MS5 sequential co-culture system was used to differentiate hPSCs to the B cell lineage (Figure 4A) (Carpenter et al., 2011; Vodyanik et al., 2005). Co-cultured hematopoietic cells were analyzed after 10 days of OP9 co-culture (D10) and after further co-culture in lymphoid conditions on MS5 (approximately D31). Pro and preB cells were readily produced from MIFF3 hIPSCs (Figure 4B). Differentiation of H1 hESCs performed similarly to that seen from MIFF3 hIPSCs (Figure S3A) (Carpenter et al., 2011). An IL-7R progenitor (CD19^{-}CD34^{+}CD45RA^{+}IL-7R^{+}) of

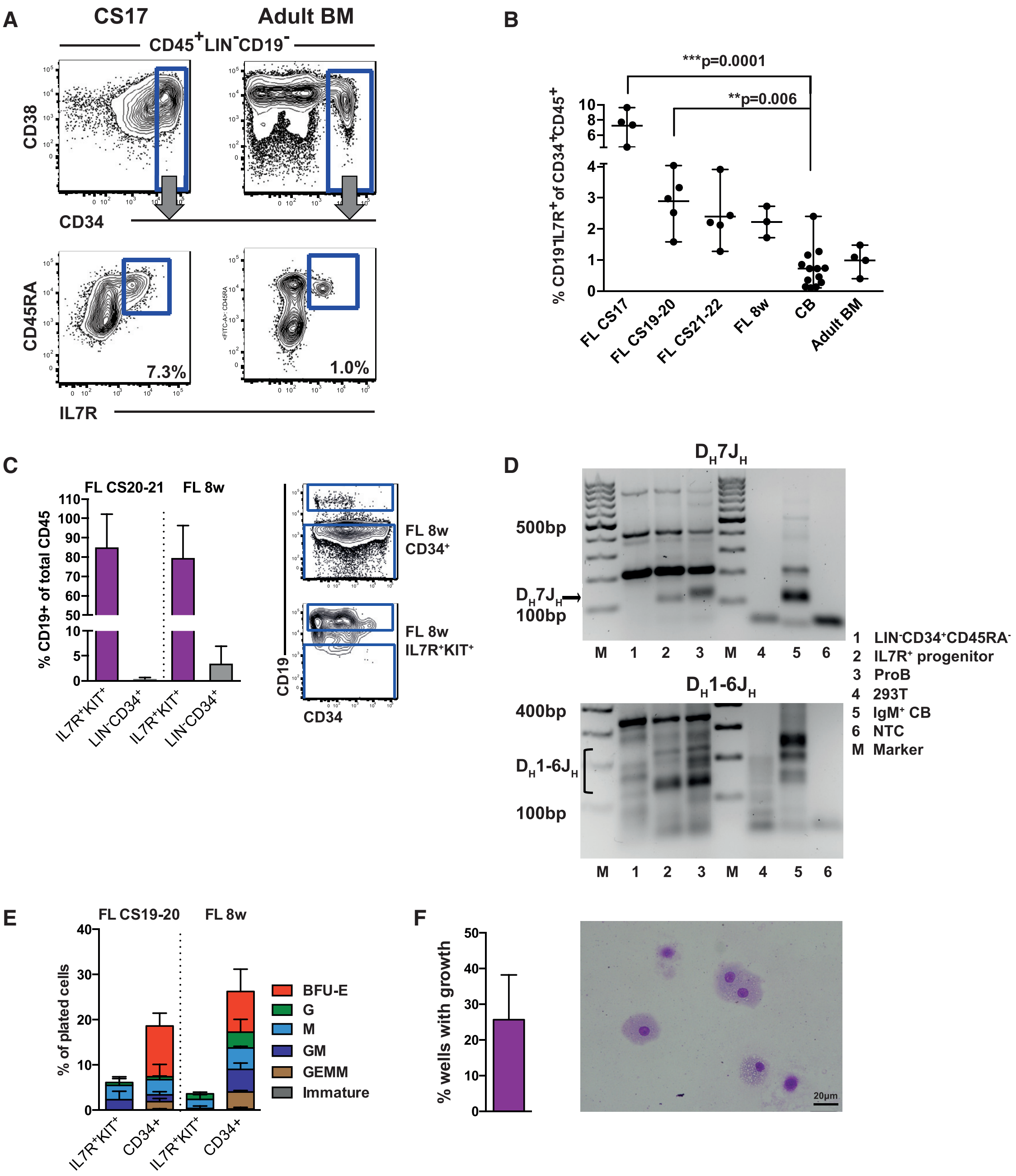

Figure 2. Identification of a CD19⁻IL-7R⁺ Progenitor

(A) Fluorescence-activated cell sorting (FACS) analysis of CS17 FL showing CD19⁻IL-7R⁺ progenitor co-expressing CD34 and CD45RA. Adult BM (right) is shown for comparison. Cells were gated CD45⁺LIN⁻CD19⁻. Further gating as indicated. Mean percentage of total CD45⁺CD34⁺ cells.

(B) Kinetics of IL-7R progenitor emergence during development as percentage of total CD45⁺CD34⁺ cells. Each dot represents one biological sample. Mean with range. Statistical comparison versus CB control.

(C) B cell potential of IL-7R⁺KIT⁺ FL progenitors was tested by co-culture on MS-5 for 2 weeks. Percentage of CD19⁺ B cells of total CD45⁺ cells is shown (left) with representative FACS analysis (right). CD34⁺ cells were used as control. Mean ± SD, n = 2–3.

(D) Agarose gel of *IGH* DJ rearrangements in IL-7R⁺ FL progenitor. Top: D_H7J_H recombination assay. Bottom: D_H1-$6J_H$ recombination assay. Label for lanes 1–6 is shown to the right. Marker lanes (M). Predicted D_H7J_H recombination between 100 and 130 bp. Predicted D_H1-$6J_H$ rearrangements between 110 and 290 bp, n = 1 (van Dongen et al., 2003).

(E) GM and erythroid potential of IL-7R⁺KIT⁺ FL progenitor in semi-solid media. Data show distribution of colony types as percentage of plated cells versus CD34⁺ cells as controls. Mean ± SD, n = 2 per developmental stage.

(F) Myeloid potential of IL-7R⁺KIT⁺ FL progenitor in liquid culture (20–40 cells/well). Graph shows percentage of wells expanded to >50 cells (left). Cytospin of macrophages generated from FL IL-7R⁺KIT⁺ progenitor (right). Mean ± SD, n = 3.

See also Figure S2.

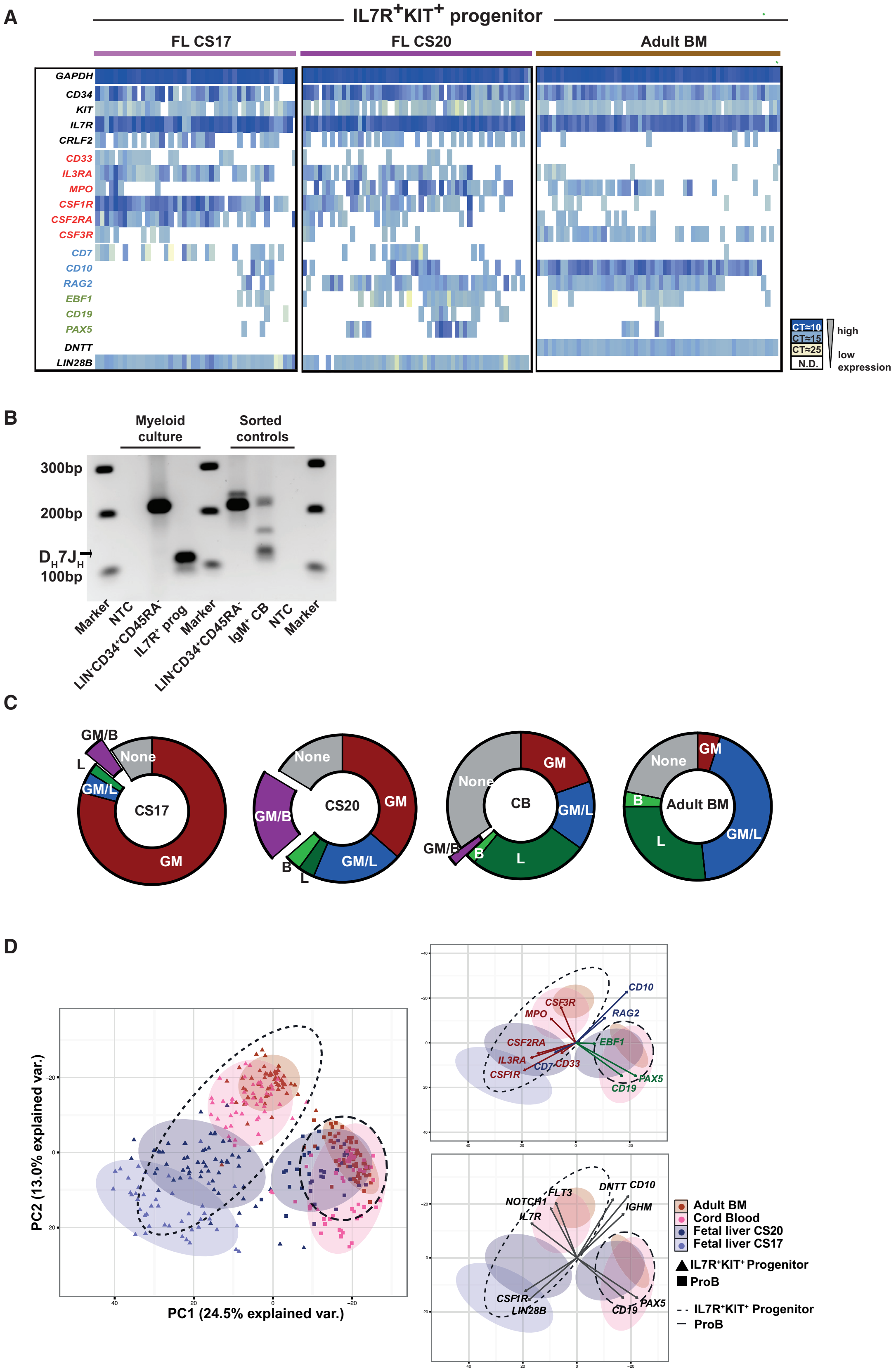

(legend on next page)

similar phenotype to the IL-7R progenitor observed in FL was also identifiable at D31 (Figures 4B and S3A). This progenitor was also seen as early as D10, significantly before the emergence of B cells or the addition of lymphoid cytokines. Population-level qPCR gene expression analysis from D31 confirmed the lymphoid nature of the D31 IL-7R progenitor and proB cells, and expression of *LIN28B* confirmed their fetal character (Figure 4C, sorting strategy Figure S3A) (Yuan et al., 2012). As in the FL, the IL-7R progenitor gave robust B cell output on MS5 co-culture (>70% of $CD45^+$ cells) and macrophage colonies in semi-solid medium, but with no erythroid colonies (Figures S3B and S3C). We tested the clonal macrophage potential of single-cell hPSC-derived IL-7R progenitor in liquid culture, showing a robust cloning frequency equivalent to that from the primitive $CD34^+CD45RA^-$ controls (13% versus 20%) (Figure S3D).

We next used RNA sequencing (RNA-seq) to globally measure the similarity between hPSC- and FL-derived B cell hierarchies. We examined both IL-7R progenitor and proB cell populations, and used $LIN^-CD34^+CD38^-CD45RA^-$ cells as more primitive controls (Doulatov et al., 2012). We controlled for any variation between individual specimens by analyzing biological replicates of each population. The data were analyzed by PCA as a measure of the overall similarity between samples. We first constructed a PCA map to compare B lymphopoiesis in adult BM with FL. As expected, PCA clearly separated the populations into three distinct clusters based on differentiation stage (PC1, 28.5% explained variance) (Figure 4D). Furthermore, the differentiation hierarchies from adult and fetal samples were also clearly separable based on developmental origin (PC2, 16.2% explained variance). Remarkably, interpolating the hPSC lymphoid hierarchy (hIPSC [MIFF3] as well as hESCs [H1]) onto this plot revealed that the hPSC clusters closely mapped with those seen from human FL and clearly separate to those from adult BM (Figure 4D). This interpolation strategy was used to mitigate distortion of the PCA by culture-related signatures; however, a PCA, calculated using primary and hPSC samples, produced similar results, with hPSCs further separated from adult BM (Figure S3E). Collectively these global gene expression profiles support the notion that *in vitro* hPSC differentiation closely reflects that seen in FL, with both being distinct from adult. Since cALL is initiated in fetal tissues, this indicates that hPSCs provide a developmentally relevant model of early embryonic human B lymphopoiesis in which to model the pre-leukemic initiation of cALL.

Lastly, we used single-cell qPCR to explore whether the hPSC system exhibits the transition from myeloid to lymphoid programming seen in the human FL IL-7R progenitor compartment. The early D10 IL-7R progenitor exhibited a strong and relatively uniform myeloid signature similar to the fetal CS17 IL-7R progenitor. By D31 there was a mixture of myeloid-, lympho-myeloid-, and B lineage-primed cells (Figures 4E and S3F). Analysis by PCA showed that the early D10 hPSC-derived progenitors overlapped closely with CS17 FL, whereas D31 IL-7R progenitor overlapped both the CS17 and CS20 FL progenitors, reflecting the mix of myeloid- and lympho-myeloid-primed cells seen in Figure 4E (Figure 4F). The hPSC proB cells clustered away from the IL-7R progenitor, partially overlapping the territory occupied by the CS20 FL proB cells and distinct from adult BM proB cells (Figure 4F).

Thus, the hPSC-derived IL-7R progenitor and proB cells resemble the FL cells both functionally and transcriptionally, and the hPSC cultures appear to recapitulate the myeloid to lympho-myeloid transition seen in the IL-7R progenitor during embryonic development. As such, this system offers an experimentally tractable system in which to model the pre-leukemic phase of ALL initiated by ETV6-RUNX1 in early human ontogeny.

An hIPSC Model of ETV6-RUNX1 Shows a Block in B Lineage Commitment

Since ETV6-RUNX1's impact is likely dose-dependent, we used a homologous recombination knockin approach to ensure appropriate regulation and levels of ETV6-RUNX1. The cDNA encoding *RUNX1* exons II–VIII was introduced into the native *ETV6* locus of MIFF3 hIPSCs at the breakpoint region by CRISPR Cas9*D10A*-directed homologous recombination (Figure 5A). ETV6-RUNX1 is linked to an mVenus reporter by a self-cleaving 2A peptide. hPSC clones were genotyped by Southern blot hybridization and tested for normal karyotype and ongoing expression of pluripotency markers (Figures S4A–S4E). Importantly, western blot analysis confirmed expression of the ETV6-RUNX1 protein at levels equivalent to that seen in the ETV6-RUNX1-expressing cell line REH (Figure S4F).

ETV6-RUNX1-expressing clones produced either no or low numbers of immunophenotypically defined $CD19^+$ proB and preB cells relative to control cultures, although an IL-7R progenitor could be detected at numbers similar to that in controls (Figures 5B and 5C). This suggests that ETV6-RUNX1 affects a partial block in B cell differentiation at the IL-7R progenitor to proB cell transition. To directly attribute the cellular and molecular phenotypes seen in the knockin cells to ETV6-RUNX1 expression, we cloned two further control lines where the *RUNX1* knockin cassette had been removed using TAT-CRE.

Figure 3. The IL-7R Progenitor Transitions from a Myeloid to a Lymphoid Gene Expression Signature during Early Embryonic Development

(A) Single-cell qPCR analysis of $IL\text{-}7R^+KIT^+$ progenitor from different developmental stages; FL CS17 (left), FL CS20 (middle), and adult BM (right). Each column represents a single cell, colored by cycle threshold (CT) value. Only cells expressing *GAPDH* are shown. Gene sets: red, myeloid; blue, lymphoid; green, B lineage related; 44–60 cells and n = 3 per developmental stage.

(B) Agarose gel of D_H7J_H rearrangement in myeloid cells derived from myeloid liquid culture of CS20 $IL\text{-}7R^+$ progenitors (also sorted positive for KIT), n = 1; marker lanes (M) 1 kb+ (Invitrogen). Predicted D_H7J_H recombination band 100–130 bp (van Dongen et al., 2003).

(C) Co-expression of lineage-associated genes in $IL\text{-}7R^+KIT^+$ progenitor based on single-cell qPCR. Cells are considered myeloid (GM), lymphoid (L), or B-primed (B) if expressing two genes representative of that lineage, as defined in Figure 3A. Co-expression of two or more of each of the GM and L and or B genes are labeled GM/L and GM/B, respectively. Only cells expressing *GAPDH* and *IL7R* were analyzed.

(D) PCA from single-cell qPCR data of $IL\text{-}7R^+KIT^+$ progenitor (triangles) and proB cells (squares) from FL (CS17 [progenitor only] and CS20), CB, and adult BM. Each dot represents a single cell. Right graphs show the direction and magnitude of eigenvectors contributing to separation for lineage-associated and most differentially expressed genes, respectively. Thirty-six genes were used for the PCA, n = 2–3 per developmental stage. Ellipses represent region of 90% confidence for each cellular compartment.

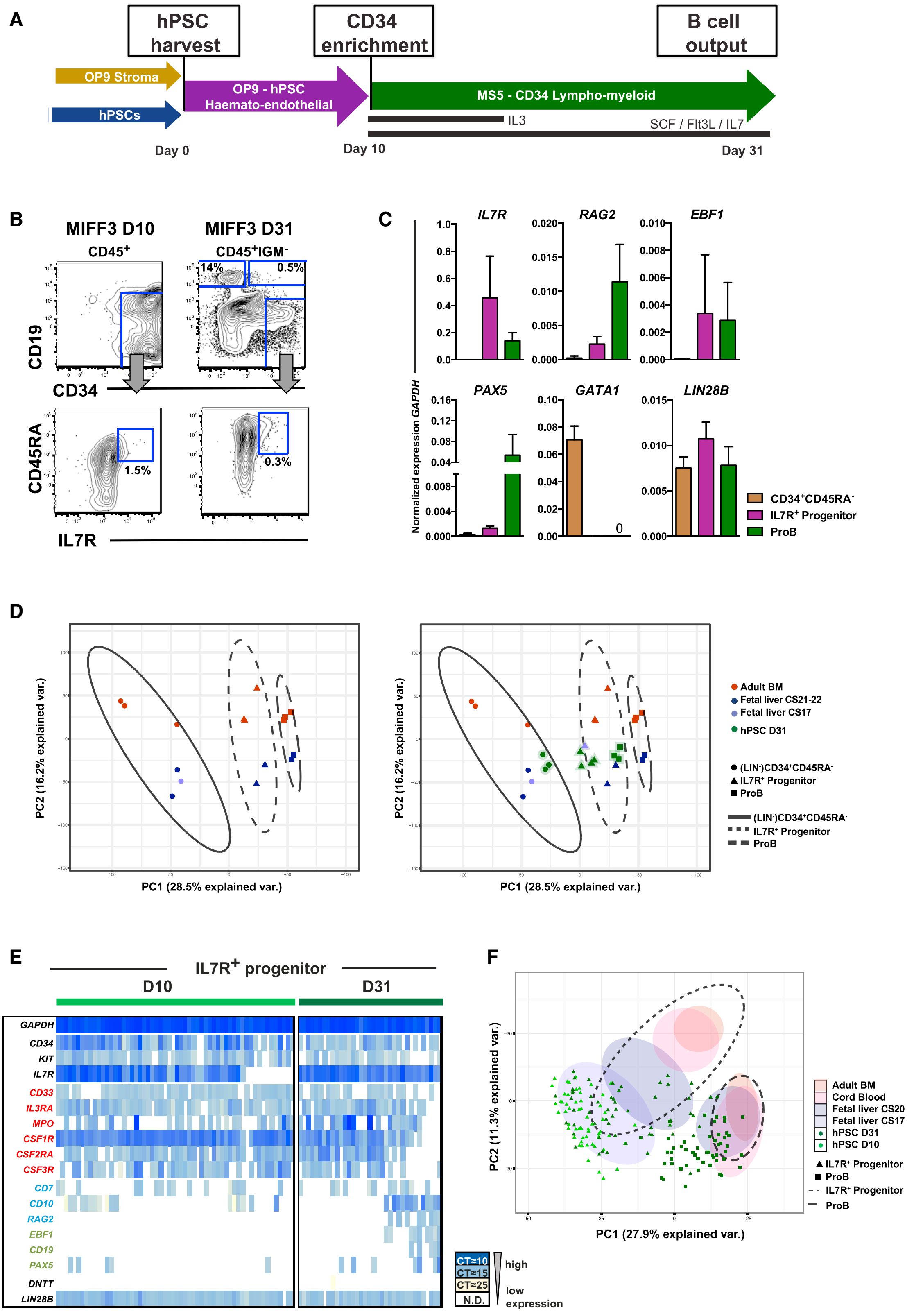

Figure 4. Lymphoid Commitment in hPSC Culture Recapitulates Human Fetal B Lymphopoiesis

(A) hPSC *in vitro* B cell differentiation protocol. hPSCs are harvested onto a layer of overgrown OP9 and co-cultured for 10 days. After CD34 enrichment, cells were either analyzed (D10) or co-cultured for an additional 21 days on MS-5 in the presence of lymphoid cytokines as indicated (D31) (Carpenter et al., 2011).

(legend continued on next page)

This approach left the PGK-NeoR cassette *in situ*, controlling for any confounding effects of this part of the construct (Strathdee et al., 2006; Zhu et al., 2015). These reverted clones produced proB and preB cells at the expected frequency (Figures 5B and 5C). We tested whether cells expressing ETV6-RUNX1 exhibited any clonal advantage over wild-type by competitive co-culture of wild-type MIFF3 and ETV6-RUNX1 knockin CD34$^+$ cells from D10 of culture (Figure 5D). By days 29–31 of B lineage-differentiating conditions, wild-type CD34$^-$CD19$^+$ preB cells dominated (98%, *p < 0.05), whereas the more primitive ETV6-RUNX1$^+$ IL-7R progenitors outcompeted their wild-type counterparts (83%, *p < 0.05) (Figure 5E). Overall, these data suggest that ETV6-RUNX1 affects a partial block in early B cell lineage restriction at the IL-7R progenitor to proB cell transition, resulting in a relative expansion of the IL-7R progenitor.

ETV6-RUNX1 "proB" Cells Exhibit Multilineage Priming and Potential

To explore the point in B cell differentiation that ETV6-RUNX1 is having its first transcriptional impact, we performed global RNA-seq on the ETV6-RUNX1 B lymphoid hierarchy, including the small numbers of ETV6-RUNX1 proB cells that escaped the differentiation block. Overlaying these data on the PCA calculated using primary human cells showed that ETV6-RUNX1$^+$ primitive CD34$^+$CD45RA$^-$ and IL-7R progenitor compartments clustered with the IPS-derived cells at the global level. In contrast, the ETV6-RUNX1-expressing CD19$^+$ "proB" cells clustered with CS17 and control hPSC-derived IL-7R progenitor, rather than control hPSC-derived proB cells (Figure 6A, left). We used gene set enrichment analysis (GSEA) to confirm that the ETV6-RUNX1$^+$ "proB" cells were indeed more mature than the ETV6-RUNX1$^+$ IL-7R progenitor, showing marked enrichment for known proB cell genes (Laurenti et al., 2013) (normalized enrichment score −2.07, false discovery rate q = 0.00) (Figure S5A). ProB cells derived from the reverted ETV6-RUNX1$^-$ hPSC lines clustered with their wild-type counterparts, demonstrating that this shift in programming is attributable to the presence of ETV6-RUNX1 (Figure 6A, right).

We investigated to what extent genes, known to be altered in B-ALL, also changed in our IPS ETV6-RUNX1 model. The erythroid-associated gene *EPOR*, known to be upregulated in ETV6-RUNX1$^+$ B-ALL, was significantly increased in the IPS ETV6-RUNX1 proB cells (Torrano et al., 2011) (Figure 6B). We found the lymphoid- and B-related genes *IL7R*, *EBF1*, and the B cell tumor suppressor *PAX5*, to be significantly downregulated in the ETV6-RUNX1$^+$ proB cells, whereas more myeloid genes (*CSF1R*, *SPI1*, and *CEBPA*), and those expressed in more primitive cells (*FLT3* and *MPL*), were upregulated (Abdelhaleem, 2007; Iijima et al., 2000; Tijchon et al., 2013) (Figure 6B). Target genes of the B cell transcription factor, STAT5, activation of which is known to co-operate with haploinsufficiency of PAX5 and EBF1 in initiating ALL (Heltemes-Harris et al., 2011), were markedly upregulated in ETV6-RUNX1 proB cells, as shown by GSEA (Figure 6C). These observations indicate that the hIPSC ETV6-RUNX1$^+$ proB cells share significant molecular similarities to those seen in cALL. As a first hit in our model, ETV6-RUNX1 undermines B lineage identity of the pre-leukemic proB cells and facilitates the co-expression of signaling and transcriptional regulators associated with more primitive and myeloid lineages.

To gain a broader appreciation of how ETV6-RUNX1 affects B lineage commitment we used GSEA of the RNA-seq data interrogating HSC-, megakaryocyte/erythroid (MkE)-, myeloid-, and B cell-associated signatures (Laurenti et al., 2013) (Figure 6D). The ETV6-RUNX1$^+$ "proB" cells were highly enriched for HSC, MkE, and myeloid genes compared with hPSC-derived controls. Although the early B cell signature was less well developed in ETV6-RUNX1 "proB" cells than in controls, they nevertheless displayed components of B cell identity. We analyzed this further by single-cell qPCR to assess the extent of heterogeneity and co-expression of lineage-affiliated genes in the ETV6-RUNX1-expressing "proB" cells. ETV6-RUNX1 "proB" cells had widespread co-expression of a B-myeloid signature, significantly enhanced relative to that from both MIFF3 and reverted hPSC-derived proB cells (Figures 6E, S5B, and S5C). This suggests that expression of ETV6-RUNX1 imposes or maintains multilineage gene expression priming characteristic of primitive cells, which is not fully resolved as the cells initiate B lineage commitment.

The increased expression of myeloid surface markers prompted us to investigate if the ETV6-RUNX1 "proB" cells could survive in liquid culture supplemented with myeloid cytokines. We found a significant increase in small colonies after 2 weeks of culture compared with wild-type proB cells, demonstrating an increased survival of these pre-leukemic cells (Figure 6F). In addition, a proportion (8.8%) of the ETV6-RUNX1 "proB" cell clones differentiated into large macrophage-containing colonies, demonstrating that ETV6-RUNX1$^+$ "proB" cells exhibit an unexpected capacity for lineage plasticity (Figures 6G and 6H).

(B) Differentiated MIFF3 hPSCs were analyzed for CD19$^+$ B cells (top) and CD19$^-$IL-7R$^+$ progenitor (bottom) at D10 (left) and D31 (right). Cells were gated CD45$^+$ or CD45$^+$IGM$^-$; further gating as indicated. Mean percentages of CD45$^+$ cells, n = 5–7.

(C) qPCR of D31 hPSCs. CD34$^+$CD45RA$^-$, IL-7R$^+$ progenitor, and CD34$^+$CD19$^+$ proB cells were analyzed for expression of lymphoid (*IL7R*, *RAG2*, *EBF1*, and *PAX5*), megakaryocytic/erythroid (*GATA1*), and fetal-specific (*LIN28B*) genes. Data are normalized to *GAPDH*, mean ± SD, n = 2–3.

(D) PCA of RNA-seq data. Left: PCA based on primary cells only; LIN$^-$CD34$^+$CD45RA$^-$, IL-7R$^+$ progenitor, and proB cells from adult BM (n = 3) and FL (CS21-22, n = 2). Right: hPSC-derived cells and the early CS17 FL IL-7R$^+$ progenitor (outlier) were placed on the PCA calculation shown to the left. hPSCs (MIFF3 and H1) are from D31 (CD34$^+$CD45RA$^-$, IL-7R$^+$ progenitor, and proB cells) of differentiation. PCA1 and PCA2 are shown and each dot represents one sample. The ellipses show the 90% density function for that cell type.

(E) Single-cell qPCR analysis of IL-7R$^+$ progenitor from D10 and D31 of differentiation (MIFF3). Each column represents a single cell. Colored by CT value. Only cells expressing *GAPDH* are shown. Genes in red are myeloid, blue are lymphoid, and green are B cell genes. A total of 35–58 cells per time point, n = 2/population.

(F) PCA calculated from single-cell qPCR data from the human primary cells (Figure 3D) and differentiated hPSCs (MIFF3 and H1). hPSCs are from D10 (IL-7R$^+$ progenitor only) and D31 (IL-7R$^+$ progenitor and proB cells). Each dot represents a single cell (only hPSCs shown). Highlighted ellipses represent primary human cell clusters, 36 genes were used for the PCA, n = 2–3.

See also Figure S3.

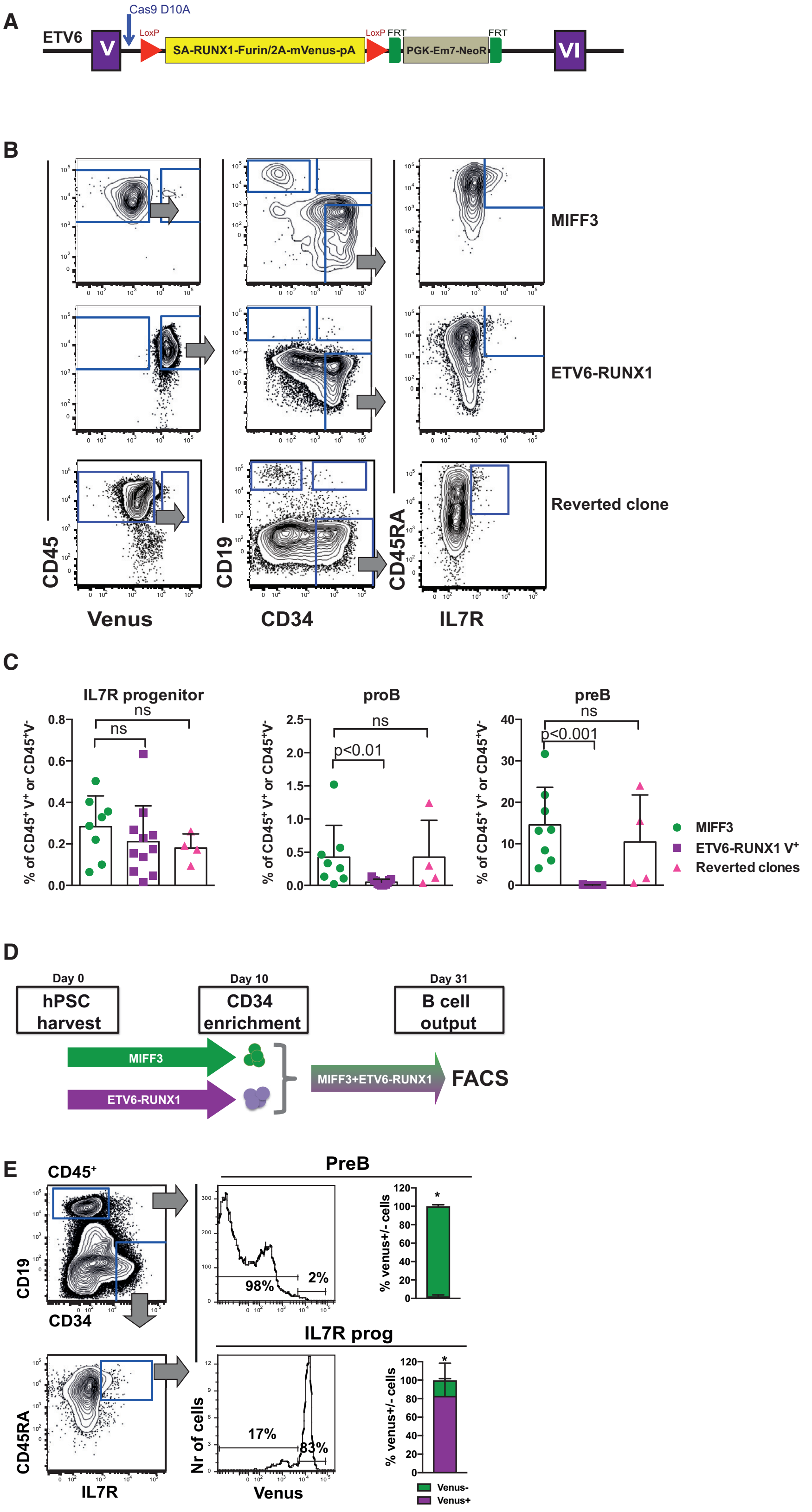

Figure 5. An hIPSC Model of ETV6-RUNX1 Shows a Block in B Lineage Commitment

(A) Genome engineering strategy. A constitutive knockin cassette, encoding a splice acceptor (SA), cDNA for *RUNX1* exons II–VIII V5 tagged at the C terminus, and linked to the mVenus fluorescent reporter by a self-cleaving furin/T2A peptide is inserted by CRISPR-directed homologous recombination toward the 5′ end of *ETV6* intron V. The cassette is flanked by *LoxP* sites and includes a 3′ triple stop/poly(A) tail followed by an FLP floxable NeoR$^+$ selection cassette.

(B) Control MIFF3 (top panel), ETV6-RUNX1 hPSCs (middle panel) and reverted ETV6-RUNX1 hPSCs (bottom panel) were cultured according to Figure 4A and analyzed by flow cytometry for Venus reporter (left), proB (CD34$^+$CD19$^+$) and preB (CD34$^-$CD19$^+$) cells (middle), and IL-7R$^+$ progenitor (right). Viable cells were gated as indicated.

(C) Frequencies of IL-7R$^+$ progenitor, proB, and preB cells in MIFF3 cells compared with ETV6-RUNX1 iPSCs and reverted ETV6-RUNX1 hPSCs analyzed at D27-31. Percentage of Venus$^-$ (MIFF3/reverted clone) or Venus$^+$ (ETV6-RUNX1 hIPSC) CD45$^+$ blood cells. Each dot represents one replicate. Only samples with 8,000 cells or more were considered. Mean ± SD. Statistics were performed using MIFF3 as control group, ns, not significant.

(D) Schematic drawing of *in vitro* competitive assay. Equal numbers of ETV6-RUNX1 and wild-type MIFF3-derived CD34$^+$ cells from D10 of OP9 co-culture were seeded onto MS5 in B differentiating conditions. The proportions of Venus$^+$ and Venus$^-$ preB cell and IL-7R progenitors were analyzed by FACS at D29-31.

(E) Differentiated MIFF3 and ETV6-RUNX1$^+$ hPSCs, grown in competition, were analyzed for CD34$^-$CD19$^+$ B cells (top) and CD19$^-$IL-7R$^+$ progenitor (bottom) at D29–D31. Cells were gated CD45$^+$; further gating as indicated. FACS plot of representative sample annotated with mean percentages values. Summary bar charts show mean ± SD, *p < 0.05, n = 4.

See also Figure S4.

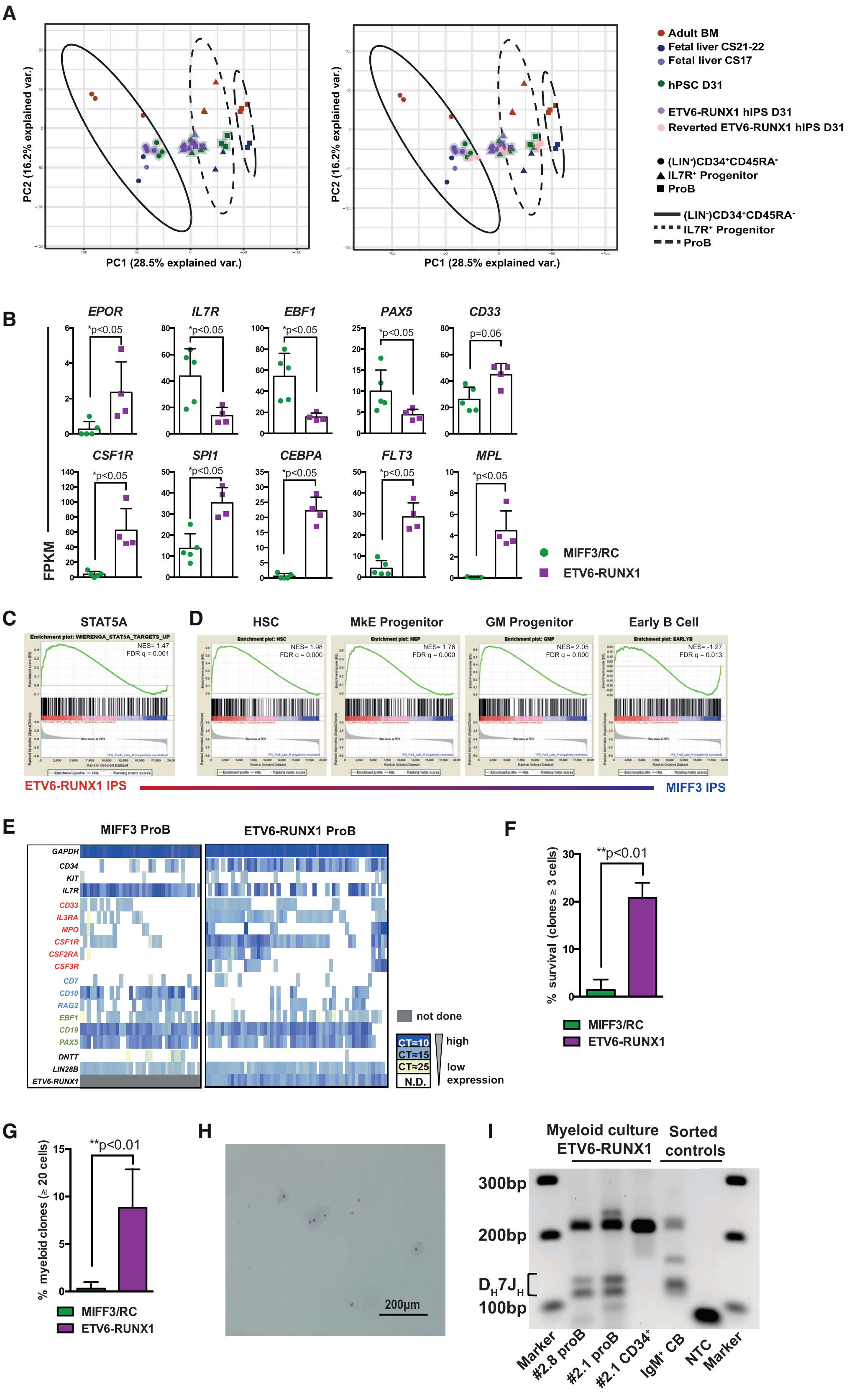

(legend on next page)

We confirmed the lymphoid origins of these myeloid colonies by demonstrating *IGH* DJ recombination in the myeloid cells (Figure 6I).

These data are consistent with ETV6-RUNX1 functioning as a first-hit mutation by specifically dysregulating a developmentally restricted myeloid to lymphoid transition; this partially blocks B lineage commitment resulting in pre-leukemic "proB" cells that retain the characteristics of a primitive fetal lympho-myeloid progenitor.

DISCUSSION

Our study was predicated on the hypothesis that developmental differences in human fetal B cell progenitors may provide a susceptibility to pre-leukemic initiation by ETV6-RUNX1. Herein, we report that: (1) the earliest $CD19^{-}IL\text{-}7R^{+}$ lymphoid progenitors in the human FL transition from a myeloid to lymphoid gene expression signature during development; (2) these transcriptional states, unique to early fetal development, can be recapitulated *in vitro* by the directed differentiation of hPSCs; and (3) a hPSC model of ETV6-RUNX1 specifically affects this developmental transition, resulting in expansion of the $CD19^{-}IL\text{-}7R^{+}$ compartment, a partial block in B lineage commitment and the production of proB cells with aberrant myeloid gene expression signatures and potential. Our results are consistent with the notion that the changes in transcriptional states occurring in hematopoietic progenitors during early human B cell ontogeny provide a developmental susceptibility to pre-leukemic initiation by ETV6-RUNX1.

Despite extensive efforts to model infant and childhood B-ALL, including ETV6-RUNX1, in mice (Schindler et al., 2009; van der Weyden et al., 2011), these have so far provided limited insight into the earliest stages of pre-leukemic initiation and failed to faithfully recapitulate the lymphoid disease, highlighting the need to explore developmentally relevant human model systems. We have addressed this by exploring the earliest stages of human fetal B lymphopoiesis, the developmental stage from which cALL is presumed to initiate. While examining the functional effects of ETV6-RUNX1 in primary human fetal material is technically infeasible, we hypothesized that hPSCs might provide a platform in which to model human fetal lymphopoiesis. Therefore, in parallel, we have characterized the nature of B lymphopoiesis emanating from hPSCs *in vitro*, aiming (1) to understand the extent to which this model system reflects fetal, as opposed to adult, B lineage hierarchies, thus providing a tractable experimental model to analyze the molecular events underlying B lineage commitment *in utero*, and (2) explore how these developmental processes confer susceptibility to oncogenes that act *in utero*, such as ETV6-RUNX1.

Strikingly, the first B cells that emerge during human development are distinct from their adult compartments in their extensive expression of IL-7R, allowing us to identify a $CD19^{-}IL\text{-}7R^{+}$ lymphoid progenitor. At CS17, where B cells first emerge, the $CD19^{-}IL\text{-}7R^{+}$ progenitor expresses a predominantly myeloid gene expression program, whereas by CS20, where B cell production is more robust, the IL-7R progenitor exhibits a lympho-myeloid program, which single-cell gene expression analysis demonstrates as being co-resident within individual cells. This B-myeloid co-expression occurs even in the presence of B cell master regulators, notably the transcription factor PAX5. Consistent with this molecular analysis, the $CD19^{-}IL\text{-}7R^{+}$ compartment produced both B and myeloid cells at the population level, and myeloid cells derived from the IL-7R progenitor harbored *IGH* DJ rearrangements, confirming their origins from a RAG^{+} lymphoid progenitor (Figure 3B). This mix of myeloid and lympho-myeloid programming distinguishes the early fetal IL-7R compartment from the $IL\text{-}7R^{+}$ progenitors previously reported in adult and later fetal developmental stages, which have been characterized as lymphoid progenitors (Roy et al., 2012; Ryan et al., 1997).

The developmental nature of *in vitro* hPSC-derived hematopoietic progenitors is an area of considerable controversy (Slukvin, 2013). T and, where achieved, B lymphoid output is generally considered evidence of "definitive" hematopoiesis, but reproducible derivation of engraftable dHSCs from hPSCs has been elusive, raising the possibility that B lymphopoiesis from hPSCs

Figure 6. ETV6-RUNX1 "proB" Cells Exhibit Multilineage Priming and Potential

(A) RNA-seq data from ETV6-RUNX1-expressing iPSCs overlaid (haloed) on the existing PCA map calculated from primary samples (Figure 4D). Control hPSC-derived data (haloed) are shown for comparison (left). PCA also including reverted ETV6-RUNX1 IPS (pink) (right). PC1 and PC2 are shown; each dot represents one sample. The ellipses show the 90% density function for that cell type. Two different ETV6-RUNX1 cell lines (no. 2.1 and no. 2.8) used in thre experiments. Reverted clone, n = 2, in one experiment.

(B) Comparison of gene expression in MIFF3/reverted ETV6-RUNX1 (RC) (green) and ETV6-RUNX1 hIPSC (purple) proB cells. Each dot represents one sample. Bars show mean fragments per kilobase of transcript per million fragments mapped (FPKM) value ± SD, n = 4–5.

(C) Gene set enrichment analysis (GSEA) of STAT5A target gene expression comparing ETV6-RUNX1 hIPSC (red) with control proB cells at D31 of differentiation. NES, normalized enrichment score. FDR, false discovery rate.

(D) GSEA as in (C). Lineage affiliations of gene sets used are indicated above (Laurenti et al., 2013).

(E) Single-cell qPCR data of control MIFF3 and $ETV6\text{-}RUNX1^{+}$ proB cells from D31. Each column represents a single cell. Colored by CT value. Genes labeled in red are myeloid, blue are lymphoid, and green are B cell genes. Only cells expressing *GADPH* and *ETV6-RUNX1* are shown. A total of 37–56 cells investigated per population, n = 2–3.

(F) Survival of MIFF3/reverted ETV6-RUNX1 (RC) (green) and ETV6-RUNX1 Venus $(V)^{+}$ hIPSC (purple)-derived single proB cells ($CD34^{+}CD19^{+}$) grown in liquid culture supplemented with myeloid cytokines (percentage of wells with ≥ 3 cells at 14 days). Mean ± SD, n = ETV6-RUNX1 = 4; MIFF3/reverted = 6, in two experiments.

(G) Myeloid differentiation potential MIFF3/reverted ETV6-RUNX1 (RC) (green) and ETV6-RUNX1 Venus $(V)^{+}$ (purple)-derived single proB cells ($CD34^{+}CD19^{+}$) grown in liquid culture supplemented with myeloid cytokines (percentage of wells with ≥ 20 cells at 14 days). Mean ± SD, n = ETV6-RUNX1 = 4; MIFF3/reverted = 6, in two experiments.

(H) Cytospin of macrophages generated from $ETV6\text{-}RUNX1^{+}$ proB cells.

(I) Agarose gel of D_H7J_H rearrangements in myeloid cells derived from liquid culture of ETV6-RUNX1 proB cells (clones no. 2.1 and no. 2.8) and primitive $ETV6\text{-}RUNX1^{+}$ $CD34^{+}$ cells. Marker lanes (M) 1 kb+ (Invitrogen). D_H7J_H recombination predicted band 100–130 bp (van Dongen et al., 2003). See also Figure S5.

is not definitive in nature (Kennedy et al., 2012). Comparison of hPSC derivatives with fetal counterparts provides a crucial point of reference to understand the developmental nature of hPSC-derived B lymphopoiesis. *In vitro* differentiation of hPSCs produces an IL-7R progenitor with similar functional capacity to that seen in the early FL. Importantly single-cell qPCR analysis at different time points during *in vitro* differentiation demonstrated a transition from myeloid to lymphoid programming similar to that seen in the fetus, suggesting that the hPSC system recapitulates this early developmental lineage progression. Indeed, our comparison of global transcriptional profiles demonstrates clear transcriptional differences separating fetal and adult B lymphopoiesis and reveals that the hPSC-derived hierarchy shares a remarkable degree of overlap with the developmental signature of FL. Together our data indicate that hPSCs provide a good model to understand how ETV6-RUNX1 dysregulates early human fetal lymphopoieis.

In this developmental context, ETV6-RUNX1 initiates a partial differentiation block at the IL-7R progenitor to proB cell transition (Figures 5B–5E and 6A). In contrast, murine models using viral overexpression systems have previously shown that ETV6-RUNX1 blocks B cell differentiation at the proB (Tsuzuki and Seto, 2013; Tsuzuki et al., 2004) or preB cell (Morrow et al., 2004) stage. Although *in vitro* differentiation of hPSCs to the B lineage can be technically variable, by producing multiple knockin cell lines that demonstrate similar phenotypes and by sub-cloning further lines with the *RUNX1* knockin cassette removed, we have controlled for variability in hPSC culture and differentiation. Importantly, the knockin approach used here provides a more authentic recapitulation of the timing and level of ETV6-RUNX1 expression than has been possible by viral transgenesis (Tsuzuki and Seto, 2013). The partial nature of this differentiation block is consistent with the identification of ETV6-RUNX1^{+} B cells in pre-leukemic CBs (Mori et al., 2002) and may explain the requirement for recurrent second-hit mutations targeting other lymphoid master regulators such as PAX5.

The precise mechanism by which ETV6-RUNX1 promotes this differentiation block remains unclear. Due to the limiting cell numbers available we were unable to characterize the binding targets and epigenetic changes induced by ETV6-RUNX1, or explore how the epigenetic state of the fetal IL-7R progenitor interfaces with ETV6-RUNX1. However, as protocols for assaying chromatin immunoprecipitation and accessibility are further refined we think that our model will provide a tractable platform for transcription factor network analysis of relevant cellular compartments.

As with previous models, expression of ETV6-RUNX1 in hPSC-derived B lineage progenitors does not result in transformation to overt leukemia. Consistent with this we have not seen engraftment of ETV6-RUNX1 progenitors, nor their control counterparts, upon transplantation into the livers of newborn NSG mice (data not shown). However, the potency of the leukemic phenotypes observed needs to be placed in the context of the low penetrance of ETV6-RUNX1 as an oncogene; thus, of the 1% of children born harboring this mutation only 1% progress to leukemia, and those with the pre-leukemia have no discernible phenotype and only modest pre-leukemic clonal size (Mori et al., 2002). This notwithstanding, expression of ETV6-RUNX1 disrupts the myeloid to lymphoid transition seen in the IL-7R progenitor compartment during development, impairing B lineage commitment and resulting in a relative expansion of ETV6-RUNX1^{+} IL-7R progenitors (Figures 5B–5E) providing a plausible pre-leukemic impetus.

An important effect of this differentiation block may be to arrest B lineage progenitors at a stage susceptible to the acquisition of second-hit mutations. RAG recombinase has been implicated in mediating the majority of second-hit mutations in ETV6-RUNX1 ALL, and is expressed by both proB cells and the majority of IL-7R progenitor cells from CS20 (Papaemmanuil et al., 2014). Importantly, sequencing studies have indicated that the cell of origin in cALL harbors DJ_H rearrangements, a feature we have seen in both the earliest emerging fetal proB and IL-7R progenitor compartments. These results are consistent with a model whereby the expanded ETV6-RUNX1^{+} IL-7R progenitor sustains a pool of RAG-expressing first-hit cells, although it is uncertain whether the second-hit mutations occur within the IL-7R progenitor population itself, or its transcriptionally abnormal B lineage progeny. Thus, while the IL-7R progenitor compartment provides a strong candidate cell of origin for ETV6-RUNX1 pre-leukemia, important questions remain regarding: (1) in which cellular compartments transformation to frank leukemia occurs; and (2) how secondary mutations further disrupt B cell commitment in a genotype and developmental stage-specific manner. These may be explored by the generation for further ETV6-RUNX1 expressing hPSC sub-clones that sequentially model the impact of co-operative second-hit mutations in relevant B lineage compartments.

The ETV6-RUNX1^{+} “proB” cells that did differentiate exhibited markedly abnormal gene expression characterized by co-expression of primitive HSC and myeloid genes. The resultant co-expression of conflicting signaling and transcriptional pathways may produce a network level lineage conflict (Banerji et al., 2013; Enver and Greaves, 1998). Such cells may have differential responses to niche signals, as well as to altered cytokine environments produced in response to infection; a proposed accelerator of leukemic progression (Ford et al., 2009; Greaves, 2006). We note the very high levels of surface IL-7R expression on fetal B cells; activating mutations in the IL-7 pathway are common second hits in pediatric ALL, suggesting that this survival pathway may have relevance in the *in utero* initiation of B-ALL (Shochat et al., 2011). IPS reprogramming and myeloid transdifferentiation of committed B cells have demonstrated the power of the B cell tumor suppressor PAX5 to both maintain B lineage identity and extinguish myeloid programming (Hanna et al., 2008; Xie et al., 2004), yet ETV6-RUNX1 facilitates multilineage priming despite expression of both *EBF1* and *PAX5* (although at lower levels than control), and so in this regard is exerting a profound effect on lineage regulation. Upregulation of *EPOR* in the IPS model also recapitulates what is seen in patients with ETV6-RUNX1^{+} ALL (Torrano et al., 2011); more broadly we see upregulation of a battery of myeloid cytokine regulators, the functional relevance of which is clearly demonstrable by the markedly increased cloning frequency and residual myeloid potential of ETV6-RUNX1^{+} “proB” cells in response to myeloid cytokines (Figures 6F–6G), a feature reminiscent of the lineage promiscuity characteristic of cALL. Activation of STAT5 signaling has been seen in ALL attributed to specific mutations in the STAT5 pathway, and is associated with adverse prognosis (Heltemes-Harris et al., 2011;

Katerndahl et al., 2017). STAT5 pathway mutations are not commonly seen in ETV6-RUNX1 ALL, but interestingly our results suggest that expression of ETV6-RUNX1 itself leads to upregulation of STAT5 target genes, thereby emphasizing the importance of STAT5 signaling in ALL in general.

Together our results suggest the hPSC ETV6-RUNX1 model provides a tractable and developmentally relevant setting for the examination of how ETV6-RUNX1 initiates pre-leukemia *in utero*. The developmentally restricted myeloid to lymphoid transition seen in the fetal IL-7R progenitor compartment offers an attractive explanation as to why ETV6-RUNX1 ALL is overwhelmingly a disease of childhood and why ETV6-RUNX1 B-ALL frequently co-expresses B and myeloid surface markers (Abdelhaleem, 2007). The transient progenitor-like nature of this population, coupled with the relatively weak oncogenic impact of ETV6-RUNX1, may explain the low rate of progression to ALL and the relatively good response of ETV6-RUNX1 ALL to chemotherapy. Whether this cellular hierarchy also confers susceptibility to non-ETV6-RUNX1-associated ALL is an exciting, but as yet untested possibility. Further analysis of this hPSC-based model may identify fetal-specific vulnerabilities in ETV6-RUNX1 ALL, informing new and more specific therapeutic targets, which could be tested within this tractable platform. In addition, the model will afford systematic introduction and evaluation of the secondary mutations prevalent in ETV6-RUNX1 ALL. Finally the concept that developmentally restricted target cells are the venues for the initiation of childhood diseases (Li et al., 2005) suggests that hPSCs could be more broadly used to model childhood malignancies.

STAR★METHODS

Detailed methods are provided in the online version of this paper and include the following:

- KEY RESOURCES TABLE
- CONTACT FOR REAGENT AND RESOURCE SHARING
- EXPERIMENTAL MODEL AND SUBJECT DETAILS
  - Human Cells
- METHOD DETAILS
  - Staining for Flow Cytometry and Cell Sorting
  - Quantitative Gene Expression Analysis
  - qPCR SYBR Green
  - *In Vitro* Cultures
  - DJ Rearrangement
  - Maintenance of hPSCs
  - *In Vitro* B Cell Differentiation of hPSCs
  - Genome Engineering
  - Nucleofection and Selection of hPSCs
  - Screening by Southern Blot Hybridization
  - Knockin hPSC Colony Expansion and Checking
  - Immunostaining
  - Karyotyping
  - Western Blot
  - Removal of the RUNX1 Cassette by Cre Recombinase
  - RNA-Seq
  - Bioinformatics
- QUANTIFICATION AND STATISTICAL ANALYSIS
- DATA AND SOFTWARE AVAILABILITY

SUPPLEMENTAL INFORMATION

Supplemental Information includes five figures and one table and can be found with this article online at https://doi.org/10.1016/j.devcel.2017.12.005.

ACKNOWLEDGMENTS

The authors thank O. Lindvall, J. Reimer, M. Järås, J. Richter, L. Nilsson, and HDBR (www.hdbr.org Joint MRC/Wellcome Trust grant# 099175/Z/12/Z) for help with human material; P. Andrews, C. Unger, and D. Baker for generation of MIFF3 and karyotyping of knockin clones; and A. French and L. Carpenter for assistance with B cell differentiation of hPSCs and providing MS5. OP9 and Cas9D10A were kindly provided by I. Slukvin and E. Payne, respectively. C. Pina, G. May, and G. Karlsson are thanked for advice with molecular analysis, and T. Roschupkina and Y. Guo for cell sorting advice. StemFit medium was kindly gifted on test by Ajinomoto, Tokyo. C.B. was funded by a postdoctoral fellow grant from the Swedish Childhood Cancer Foundation (PDS13/005) and the Swedish Research Council (no. 2015-00135), Marie Sklodowska Curie Actions, Cofund, Project INCA (no. 600398). S.E.R. was funded by a Wellcome Trust Research Training Fellowship (100056/Z/12/Z) and a UK NIHR Academic Clinical Fellowship and Clinical Lectureship. E.L. had a Cancer Research UK-funded studentship (527631). The project was supported by The Crafoord Foundation (C.B.), The Swedish Childhood Cancer Foundation (PR2013-0101) (T.E.), the Swedish Cancer Society (CAN2013/712) (T.E.), the Hemato-Linné Grant (T.E. and S.E.W.J.), the Tobias Foundation (S.E.W.J.), a Children with Cancer Grant (520347) (T.E.), and Bloodwise (533699, D-OTH) (T.E.). This work was supported by the CRUK-UCL Centre Award (C416/A25145).

AUTHOR CONTRIBUTIONS

C.B. and S.E.R. conducted the experiments with equal contribution and are listed alphabetically. C.B. was the lead on the identification of the IL-7R progenitor in FL, and S.E.R. was the lead on hPSC modeling. E.L. contributed functional hPSC experiments. A.Z. provided assistance with sorts and analysis of fetal samples. V.A.T. and J.P.W. assisted with hPSC modeling. D.W., C.J., and J.H. performed bioinformatic analysis. J.B. and A.J.H.S. contributed to genome engineering. S.K. and E.S. advised on the research plan. C.B., S.E.R., S.E.W.J., and T.E. designed and conceptualized the research, analyzed data, and wrote the manuscript.

DECLARATION OF INTERESTS

The authors declare no competing interests.

Received: December 8, 2016
Revised: August 4, 2017
Accepted: December 1, 2017
Published: December 28, 2017

REFERENCES

Abdelhaleem, M. (2007). Frequent but nonrandom expression of myeloid markers on de novo childhood acute lymphoblastic leukemia. Exp. Mol. Pathol. *83*, 138–141.

Alpar, D., Wren, D., Ermini, L., Mansur, M.B., van Delft, F.W., Bateman, C.M., Titley, I., Kearney, L., Szczepanski, T., Gonzalez, D., et al. (2015). Clonal origins of ETV6-RUNX1(+) acute lymphoblastic leukemia: studies in monozygotic twins. Leukemia *29*, 839–846.

Anderson, K., Lutz, C., van Delft, F.W., Bateman, C.M., Guo, Y., Colman, S.M., Kempski, H., Moorman, A.V., Titley, I., Swansbury, J., et al. (2011). Genetic variegation of clonal architecture and propagating cells in leukaemia. Nature *469*, 356–361.

Banerji, C.R., Miranda-Saavedra, D., Severini, S., Widschwendter, M., Enver, T., Zhou, J.X., and Teschendorff, A.E. (2013). Cellular network entropy as the energy potential in Waddington's differentiation landscape. Sci. Rep. *3*, 3039.

Boiers, C., Carrelha, J., Lutteropp, M., Luc, S., Green, J.C., Azzoni, E., Woll, P.S., Mead, A.J., Hultquist, A., Swiers, G., et al. (2013). Lymphomyeloid

contribution of an immune-restricted progenitor emerging prior to definitive hematopoietic stem cells. Cell Stem Cell *13*, 535–548.

Bolstad, B.M., Irizarry, R.A., Astrand, M., and Speed, T.P. (2003). A comparison of normalization methods for high density oligonucleotide array data based on variance and bias. Bioinformatics *19*, 185–193.

Carpenter, L., Malladi, R., Yang, C.T., French, A., Pilkington, K.J., Forsey, R.W., Sloane-Stanley, J., Silk, K.M., Davies, T.J., Fairchild, P.J., et al. (2011). Human induced pluripotent stem cells are capable of B-cell lymphopoiesis. Blood *117*, 4008–4011.

Dorrell, C., Gan, O.I., Pereira, D.S., Hawley, R.G., and Dick, J.E. (2000). Expansion of human cord blood CD34(+)CD38(-) cells in ex vivo culture during retroviral transduction without a corresponding increase in SCID repopulating cell (SRC) frequency: dissociation of SRC phenotype and function. Blood *95*, 102–110.

Doulatov, S., Notta, F., Laurenti, E., and Dick, J.E. (2012). Hematopoiesis: a human perspective. Cell Stem Cell *10*, 120–136.

Enver, T., and Greaves, M. (1998). Loops, lineage, and leukemia. Cell *94*, 9–12.

Ford, A.M., Palmi, C., Bueno, C., Hong, D., Cardus, P., Knight, D., Cazzaniga, G., Enver, T., and Greaves, M. (2009). The TEL-AML1 leukemia fusion gene dysregulates the TGF-beta pathway in early B lineage progenitor cells. J. Clin. Invest. *119*, 826–836.

Gerr, H., Zimmermann, M., Schrappe, M., Dworzak, M., Ludwig, W.D., Bradtke, J., Moericke, A., Schabath, R., Creutzig, U., and Reinhardt, D. (2010). Acute leukaemias of ambiguous lineage in children: characterization, prognosis and therapy recommendations. Br. J. Haematol. *149*, 84–92.

Greaves, M. (2003). Pre-natal origins of childhood leukemia. Rev. Clin. Exp. Hematol. *7*, 233–245.

Greaves, M. (2006). Infection, immune responses and the aetiology of childhood leukaemia. Nat. Rev. Cancer *6*, 193–203.

Greaves, M.F., and Wiemels, J. (2003). Origins of chromosome translocations in childhood leukaemia. Nat. Rev. Cancer *3*, 639–649.

Hanna, J., Markoulaki, S., Schorderet, P., Carey, B.W., Beard, C., Wernig, M., Creyghton, M.P., Steine, E.J., Cassady, J.P., Foreman, R., et al. (2008). Direct reprogramming of terminally differentiated mature B lymphocytes to pluripotency. Cell *133*, 250–264.

Heltemes-Harris, L.M., Willette, M.J., Ramsey, L.B., Qiu, Y.H., Neeley, E.S., Zhang, N., Thomas, D.A., Koeuth, T., Baechler, E.C., Kornblau, S.M., et al. (2011). Ebf1 or Pax5 haploinsufficiency synergizes with STAT5 activation to initiate acute lymphoblastic leukemia. J. Exp. Med. *208*, 1135–1149.

Hong, D., Gupta, R., Ancliff, P., Atzberger, A., Brown, J., Soneji, S., Green, J., Colman, S., Piacibello, W., Buckle, V., et al. (2008). Initiating and cancer-propagating cells in TEL-AML1-associated childhood leukemia. Science *319*, 336–339.

Horton, S.J., Jaques, J., Woolthuis, C., van Dijk, J., Mesuraca, M., Huls, G., Morrone, G., Vellenga, E., and Schuringa, J.J. (2013). MLL-AF9-mediated immortalization of human hematopoietic cells along different lineages changes during ontogeny. Leukemia *27*, 1116–1126.

Hystad, M.E., Myklebust, J.H., Bo, T.H., Sivertsen, E.A., Rian, E., Forfang, L., Munthe, E., Rosenwald, A., Chiorazzi, M., Jonassen, I., et al. (2007). Characterization of early stages of human B cell development by gene expression profiling. J. Immunol. *179*, 3662–3671.

Iijima, K., Sugita, K., Inukai, T., Goi, K., Tezuka, T., Uno, K., Sato, H., Kagami, K., and Nakazawa, S. (2000). Expression of thrombopoietin receptor and its functional role in human B-precursor leukemia cells with 11q23 translocation or Philadelphia chromosome. Leukemia *14*, 1598–1605.

Ivanovs, A., Rybtsov, S., Welch, L., Anderson, R.A., Turner, M.L., and Medvinsky, A. (2011). Highly potent human hematopoietic stem cells first emerge in the intraembryonic aorta-gonad-mesonephros region. J. Exp. Med. *208*, 2417–2427.

Katerndahl, C.D.S., Heltemes-Harris, L.M., Willette, M.J.L., Henzler, C.M., Frietze, S., Yang, R., Schjerven, H., Silverstein, K.A.T., Ramsey, L.B., Hubbard, G., et al. (2017). Antagonism of B cell enhancer networks by STAT5 drives leukemia and poor patient survival. Nat. Immunol. *18*, 694–704.

Kennedy, M., Awong, G., Sturgeon, C.M., Ditadi, A., LaMotte-Mohs, R., Zuniga-Pflucker, J.C., and Keller, G. (2012). T lymphocyte potential marks the emergence of definitive hematopoietic progenitors in human pluripotent stem cell differentiation cultures. Cell Rep. *2*, 1722–1735.

Kim, D., Pertea, G., Trapnell, C., Pimentel, H., Kelley, R., and Salzberg, S.L. (2013). TopHat2: accurate alignment of transcriptomes in the presence of insertions, deletions and gene fusions. Genome Biol. *14*, R36.

Laurenti, E., Doulatov, S., Zandi, S., Plumb, I., Chen, J., April, C., Fan, J.B., and Dick, J.E. (2013). The transcriptional architecture of early human hematopoiesis identifies multilevel control of lymphoid commitment. Nat. Immunol. *14*, 756–763.

Li, Y.S., Hayakawa, K., and Hardy, R.R. (1993). The regulated expression of B lineage associated genes during B cell differentiation in bone marrow and fetal liver. J. Exp. Med. *178*, 951–960.

Li, Z., Godinho, F.J., Klusmann, J.H., Garriga-Canut, M., Yu, C., and Orkin, S.H. (2005). Developmental stage-selective effect of somatically mutated leukemogenic transcription factor GATA1. Nat. Genet. *37*, 613–619.

Man, N., Sun, X.J., Tan, Y., Garcia-Cao, M., Liu, F., Cheng, G., Hatlen, M., Xu, H., Shah, R., Chastain, N., et al. (2016). Differential role of Id1 in MLL-AF9-driven leukemia based on cell of origin. Blood *127*, 2322–2326.

Medvinsky, A., and Dzierzak, E. (1996). Definitive hematopoiesis is autonomously initiated by the AGM region. Cell *86*, 897–906.

Medvinsky, A., Rybtsov, S., and Taoudi, S. (2011). Embryonic origin of the adult hematopoietic system: advances and questions. Development *138*, 1017–1031.

Mootha, V.K., Lindgren, C.M., Eriksson, K.F., Subramanian, A., Sihag, S., Lehar, J., Puigserver, P., Carlsson, E., Ridderstrale, M., Laurila, E., et al. (2003). PGC-1alpha-responsive genes involved in oxidative phosphorylation are coordinately downregulated in human diabetes. Nat. Genet. *34*, 267–273.

Mori, H., Colman, S.M., Xiao, Z., Ford, A.M., Healy, L.E., Donaldson, C., Hows, J.M., Navarrete, C., and Greaves, M. (2002). Chromosome translocations and covert leukemic clones are generated during normal fetal development. Proc. Natl. Acad. Sci. USA *99*, 8242–8247.

Morrow, M., Horton, S., Kioussis, D., Brady, H.J., and Williams, O. (2004). TEL-AML1 promotes development of specific hematopoietic lineages consistent with preleukemic activity. Blood *103*, 3890–3896.

Notta, F., Zandi, S., Takayama, N., Dobson, S., Gan, O.I., Wilson, G., Kaufmann, K.B., McLeod, J., Laurenti, E., Dunant, C.F., et al. (2015). Distinct routes of lineage development reshape the human blood hierarchy across ontogeny. Science *351*, aab2116.

Papaemmanuil, E., Rapado, I., Li, Y., Potter, N.E., Wedge, D.C., Tubio, J., Alexandrov, L.B., Van Loo, P., Cooke, S.L., Marshall, J., et al. (2014). RAG-mediated recombination is the predominant driver of oncogenic rearrangement in ETV6-RUNX1 acute lymphoblastic leukemia. Nat. Genet. *46*, 116–125.

Porter, S.N., Cluster, A.S., Yang, W., Busken, K.A., Patel, R.M., Ryoo, J., and Magee, J.A. (2016). Fetal and neonatal hematopoietic progenitors are functionally and transcriptionally resistant to Flt3-ITD mutations. Elife *5*, https://doi.org/10.7554/eLife.18882.

Roy, A., Cowan, G., Mead, A.J., Filippi, S., Bohn, G., Chaidos, A., Tunstall, O., Chan, J.K., Choolani, M., Bennett, P., et al. (2012). Perturbation of fetal liver hematopoietic stem and progenitor cell development by trisomy 21. Proc. Natl. Acad. Sci. USA *109*, 17579–17584.

Ryan, D.H., Nuccie, B.L., Ritterman, I., Liesveld, J.L., Abboud, C.N., and Insel, R.A. (1997). Expression of interleukin-7 receptor by lineage-negative human bone marrow progenitors with enhanced lymphoid proliferative potential and B-lineage differentiation capacity. Blood *89*, 929–940.

Schindler, J.W., Van Buren, D., Foudi, A., Krejci, O., Qin, J., Orkin, S.H., and Hock, H. (2009). TEL-AML1 corrupts hematopoietic stem cells to persist in the bone marrow and initiate leukemia. Cell Stem Cell *5*, 43–53.

Schmieder, R., and Edwards, R. (2011). Quality control and preprocessing of metagenomic datasets. Bioinformatics *27*, 863–864.

Shochat, C., Tal, N., Bandapalli, O.R., Palmi, C., Ganmore, I., te Kronnie, G., Cario, G., Cazzaniga, G., Kulozik, A.E., Stanulla, M., et al. (2011). Gain-of-function mutations in interleukin-7 receptor-alpha (IL7R) in childhood acute lymphoblastic leukemias. J. Exp. Med. *208*, 901–908.

Slukvin, I.I. (2013). Hematopoietic specification from human pluripotent stem cells: current advances and challenges toward de novo generation of hematopoietic stem cells. Blood *122*, 4035–4046.

Strathdee, D., Ibbotson, H., and Grant, S.G. (2006). Expression of transgenes targeted to the Gt(ROSA)26Sor locus is orientation dependent. PLoS One *1*, e4.

Subramanian, A., Tamayo, P., Mootha, V.K., Mukherjee, S., Ebert, B.L., Gillette, M.A., Paulovich, A., Pomeroy, S.L., Golub, T.R., Lander, E.S., et al. (2005). Gene set enrichment analysis: a knowledge-based approach for interpreting genome-wide expression profiles. Proc. Natl. Acad. Sci. USA *102*, 15545–15550.

Swaminathan, S., Klemm, L., Park, E., Papaemmanuil, E., Ford, A., Kweon, S.M., Trageser, D., Hasselfeld, B., Henke, N., Mooster, J., et al. (2015). Mechanisms of clonal evolution in childhood acute lymphoblastic leukemia. Nat. Immunol. *16*, 766–774.

Tavian, M., and Peault, B. (2005). Embryonic development of the human hematopoietic system. Int. J. Dev. Biol. *49*, 243–250.

Tijchon, E., Havinga, J., van Leeuwen, F.N., and Scheijen, B. (2013). B-Lineage transcription factors and cooperating gene lesions required for leukemia development. Leukemia *27*, 541–552.

Torrano, V., Procter, J., Cardus, P., Greaves, M., and Ford, A.M. (2011). ETV6-RUNX1 promotes survival of early B lineage progenitor cells via a dysregulated erythropoietin receptor. Blood *118*, 4910–4918.

Trapnell, C., Williams, B.A., Pertea, G., Mortazavi, A., Kwan, G., van Baren, M.J., Salzberg, S.L., Wold, B.J., and Pachter, L. (2010). Transcript assembly and quantification by RNA-Seq reveals unannotated transcripts and isoform switching during cell differentiation. Nat. Biotechnol. *28*, 511–515.

Tsuzuki, S., and Seto, M. (2013). TEL (ETV6)-AML1 (RUNX1) initiates self-renewing fetal pro-B cells in association with a transcriptional program shared with embryonic stem cells in mice. Stem Cells *31*, 236–247.

Tsuzuki, S., Seto, M., Greaves, M., and Enver, T. (2004). Modeling first-hit functions of the t(12;21) TEL-AML1 translocation in mice. Proc. Natl. Acad. Sci. USA *101*, 8443–8448.

van der Weyden, L., Giotopoulos, G., Rust, A.G., Matheson, L.S., van Delft, F.W., Kong, J., Corcoran, A.E., Greaves, M.F., Mullighan, C.G., Huntly, B.J., et al. (2011). Modeling the evolution of ETV6-RUNX1-induced B-cell precursor acute lymphoblastic leukemia in mice. Blood *118*, 1041–1051.

van Dongen, J.J., Langerak, A.W., Bruggemann, M., Evans, P.A., Hummel, M., Lavender, F.L., Delabesse, E., Davi, F., Schuuring, E., Garcia-Sanz, R., et al. (2003). Design and standardization of PCR primers and protocols for detection of clonal immunoglobulin and T-cell receptor gene recombinations in suspect lymphoproliferations: report of the BIOMED-2 Concerted Action BMH4-CT98-3936. Leukemia *17*, 2257–2317.

Vodyanik, M.A., Bork, J.A., Thomson, J.A., and Slukvin, I.I. (2005). Human embryonic stem cell-derived CD34+ cells: efficient production in the coculture with OP9 stromal cells and analysis of lymphohematopoietic potential. Blood *105*, 617–626.

Waskow, C., Paul, S., Haller, C., Gassmann, M., and Rodewald, H.R. (2002). Viable c-Kit(W/W) mutants reveal pivotal role for c-kit in the maintenance of lymphopoiesis. Immunity *17*, 277–288.

Xie, H., Ye, M., Feng, R., and Graf, T. (2004). Stepwise reprogramming of B cells into macrophages. Cell *117*, 663–676.

Yuan, J., Nguyen, C.K., Liu, X., Kanellopoulou, C., and Muljo, S.A. (2012). Lin28b reprograms adult bone marrow hematopoietic progenitors to mediate fetal-like lymphopoiesis. Science *335*, 1195–1200.

Zhu, Z., Verma, N., Gonzalez, F., Shi, Z.D., and Huangfu, D. (2015). A CRISPR/Cas-mediated selection-free knockin strategy in human embryonic stem cells. Stem Cell Reports *4*, 1103–1111.

STAR★METHODS

KEY RESOURCES TABLE

REAGENT or RESOURCE	SOURCE	IDENTIFIER
Antibodies		
CD45RA FITC (Human primary samples)	ThermoFisher	#MHCD45RA01v/ MEM-56
CD34-FITC (IPS samples)	Biolegend	#343604 / 561
CD43 FITC (IPS samples D10)	BD	#555475 / 1G10
KIT (CD117)-PE (Human primary samples)	BD	#555714 / YB5.B8
CD19-PE (IPS samples)	Biolegend	#302254 / HIB19
CD56 PECY5 (LIN) (Human primary samples)	BD	#555517 / B159
CD3 PECY5 (LIN) (Human primary samples)	Biolegend	#300310 / HIT3a
CD8a-PECY5 (LIN) (Human primary samples)	Biolegend	#301010 / RPA-T8
CD235-PECY5 (LIN) (Human primary samples)	BD	#561776 / GA-R2 (HIR2)
CD45RA PECy7 (IPS samples)	Biolegend	#304126 / HI100
CD38-PECY7 (Human primary samples)	eBioscience	#25-0384-42 / HIT3
IGM-APC (IPS samples D31)	eBioscience	#17-9998-42 / SA-DA4
CD34-APC (Human primary samples)	eBioscience	#17-0349-42 / 4H11
CD45-A700	Biolegend	#304024 / HI30 or #368513 / 2D1
IL7R (CD127)-BV421	BD	#562436 / HIL-7R-M21
CD19-BV605 (Human primary samples)	BD	#562653 / SJ25C1
CD19 APCeF780 (IPS samples)	eBioscience	#47-0199-42 / HIB19
TRA1-60-PE (IPS)	BD	#560884 / TRA-1-60
SSEA3-AF647 (IPS)	BD	#561145 / MC-631
CD14-FITC (B readout)	Biolegend	#325604 / HCD14
CD34-FITC (B readout)	Biolegend	#343604 / 561
CD45RA-FITC (B readout)	Thermofisher	#MHCD45RA01/ MEM-56
CD19-PE (B readout)	Biolegend	#302254 / HIB19
KIT (CD117)-PE (B readout)	BD	#555714 / YB5.B8
CD33-PECy7 (B readout)	eBioscience	#25-0338-42 / WM-53
CD34-PECy7 (B readout)	Biolegend	#343516 / 581
CD38-PECY7 (B readout)	eBioscience	#25-0384-42 / HIT3
CD45RA-PECy7 (B readout)	Biolegend	#304126 / HI100
CD11b-APC (B readout)	Biolegend	#101211 / M1/70
CD34-APC (B readout)	eBioscience	#17-0349-42 / 4H11
CD45-A700 (B readout)	Biolegend	#304024 / HI30
IL7R (CD127)-BV421 (B readout)	BD	#562436 / HIL-7R-M21
NKP46-BV421 (B readout)	Biolegend	#331927 / 9E2
CD19-BV605 (B readout)	BD	#562653 / SJ25C1
CD14-BV605 (B readout)	Biolegend	#301834 / M5E2
NKP46-BV650 (B readout)	Biolegend	#331927 / 9E2
TO-PRO 1 (viability)	ThermoFisher	#T3602
7-Aminoactinomycin D (7-AAD) (viability)	Sigma Aldrich, BD	#A9400 / #51-68981E
Propidium Iodide (viability)	ThermoFisher	#P3566
IGG1 kappa - murine myelmoa (Fc-Block)	Sigma Aldrich	#M9269 / MOPC21
RUNX1 (Western) (1ug/ml)	Abcam	#ab23980
Histone H3 (Western) (1ug/ml)	Abcam	#ab1791

(*Continued on next page*)

Continued

REAGENT or RESOURCE	SOURCE	IDENTIFIER
Biological Samples		
Human fetal liver	Anonymous donation	N/A
Cord blood	Anonymous donation /Poietics	N/A / #2C-150A
Adult Bone Marrow	Anonymous donation	N/A
Chemicals, Peptides, and Recombinant Proteins		
h Monocyte- Colony Stimulating Factor (hM-CSF) Terasaki (some experiments) 25ng/ml	PeProTech	#300-25
h Granulocyte- Colony Stimulating Factor (hG-CSF) Terasaki 50ng/ml	PeProTech	#300-23
h Granulocyte Monocyte- Colony Stimulating Factor (hGM-CSF) Terasaki (only some experiments) 25-50ng/ml	PeProTech	#300-03
h interleukin 3 (hIL-3) Terasaki 50ng/ml, IPS diff 10ng/ml	PeProTech	#200-03
h FLT3 Ligand (hFLT3L) Terasaki/B/IPS diff 50ng/ml	PeProTech	#300-19
h Stem Cell Factor (hSCF) Terasaki/B/IPS diff 50ng/ml	PeProTech	#300-07
h Thrombopoietin (hTPO) Terasaki/B 50ng/ml	PeProTech	#300-18
h Thymic stromal lymphopoietin (hTSLP) B (only some experiments) 10ng/ml	PeProTech	#300-62
h interleukin 7 (hIL7), IPS diff 20ng/ml	PeProTech	#200-07
mTeSR1	Stem Cell Technologies	#85850
Matrigel (IPS)	Corning	#354277
StemFitAK (IPS)	Ajinomoto	N/A
FCS batched defined (IPS differentiation)	HyClone/Thermofisher	#SH30070.03 / #26140079
TAT-Cre	Millipore	#SCR508
Critical Commercial Assays		
MethoCult H4435	Stem Cell Technologies	#04435
Cell Direct one-step qRT-PCR Kit	ThermoFisher	#11753100
48.48 Dynamic Array IFC for Gene Expression	Fluidigm	#BMK-M-48.48
SMARTer Ultra Low input RNA Kit for Seq_v3	Clontech	#634851
Nextera XT DNA Sample Preparation Kit	Illumina	#FC-131-1096
Nextera XT Index Kit	Illumina	#FC-131-1002
NextSeq 500/550 High Output v2 kit (150 cycles)	Illumina	#FC-404-2002
Deposited Data		
RNA seq data set	This paper	ArrayExpress: E-MTAB-6382
Experimental Models: Cell Lines		
MIFF3 (RRID:CVCL_1E70)	University of Sheffield	http://hpscreg.eu/cell-line/UOSi001-B
H1	WiCell	*http://hpscreg.eu/cell-line/WAe001-A*
Oligonucleotides		
Oligonucleotides, see Table S1	IDT /Sigma/ ThermoFisher	N/A
Software and Algorithms		
PRINSEQ-lite v0.20.4	(Schmieder and Edwards, 2011)	http://prinseq.sourceforge.net/manual.html
TopHat2 v2.0.11	(Kim et al., 2013)	http://cole-trapnell-lab.github.io/cufflinks/tools/
Cufflinks v2.2.0	(Trapnell et al., 2010)	http://cole-trapnell-lab.github.io/cufflinks/tools/
Gene Set Enrichment Analysis (GSEA)	Broad Institute	http://software.broadinstitute.org/gsea/index.jsp
Prism	GraphPad	https://www.graphpad.com/scientific-software/prism/
Flowjo	N/A	https://www.flowjo.com

CONTACT FOR REAGENT AND RESOURCE SHARING

Further information and requests for resources and reagents should be directed to and will be fulfilled by the Lead Contact, Tariq Enver (t.enver@ucl.ac.uk).

EXPERIMENTAL MODEL AND SUBJECT DETAILS

Human Cells

Human FLs were donated from elective terminations of pregnancy after informed consent with the approval of the ethical review board at Lund University and permission from the Swedish National Board of Health and Welfare or was provided by the Joint MRC/Wellcome Trust (grant # 099175/Z/12/Z) Human Developmental Biology Resource (www.hdbr.org). Fetuses were staged according to Carnegie Staging (CS) and were all from first trimester of pregnancy (developmental stages CS17-8pcw; gender not established). Single cell suspension of the fetal liver was obtained through mechanical disruption. Human cord blood (CB) and adult bone marrow (BM) (healthy volunteers) were donated with the approval of the ethical review board at Lund University. CB was diluted in heparin immediately after delivery. Mononuclear cells from CB and BM were isolated using lympho-prep tubes from Medinor, AB. CB was also bought from Poietics. CB and BM samples are collected anonymously and information regarding age and gender is therefore not provided.

Pluripotent stem cells used were; mRNA induced foreskin fibroblast 3 (MIFF3) hIPS cells (University of Sheffield, http://hpscreg.eu/cell-line/UOSi001-B) and H1 ES cells (WiCell, European hESC registry (*http://hpscreg.eu/cell-line/WAe001-A*)). The Steering Committee for the UK Stem Cell approved all work on human ES cells.

METHOD DETAILS

Staining for Flow Cytometry and Cell Sorting

Single cell suspensions were blocked with Fc receptor binding myeloma antibodies and then stained with specific monoclonal antibodies listed in Key Resources Table *(human primary samples and IPS samples)*. Before analysis and cell sorting the sample was dissolved in viability dye (Key Resources Table). For cell sorting samples were sometimes enriched with CD34 isolation beads (Miltenyibiotec) before staining was performed.

Progenitors were identified according to the following surface markers; **(LIN$^-$)CD34$^+$CD45RA$^-$**: (LIN$^-$)CD19$^-$CD34$^+$(CD38$^-$)CD45RA$^-$(KIT$^+$), **IL7R$^+$(KIT$^+$) progenitor**: (LIN$^-$)CD19$^-$CD34$^+$(CD38$^+$)CD45RA$^+$IL7R$^+$(KIT$^+$); **ProB**; (LIN$^-$)CD34$^+$CD19$^+$; **PreB**; CD34$^-$CD19$^+$ or CD34$^-$CD19$^+$IGM$^-$; **CD34$^+$**: (LIN$^-$)CD19$^-$CD34$^+$. Some markers were not used on IPS cells; KIT, CD38 and the lineage markers (LIN); CD3, CD8a, CD235a, CD56; CD38 surface expression was affected by culture conditions, as previously reported (Dorrell et al., 2000), and surface KIT was affected by the addition of exogenous KIT ligand. Samples were analyzed or sorted on an LSR II, FortessaX20, BD ARIA IIu or BD FACSAria III (all from BD Biosciences) and analysis were performed using the FlowJo software.

Quantitative Gene Expression Analysis

Cells were directly sorted (single cells or 25 cells/population) into 4 μl of lysis buffer (65μM of dNTP mix (TaKaRa), 0.4% of NP40 (Sigma), 2.4mM DTT (Invitrogen), 0.5U/μL RNAseOUT (Invitrogen) in nuclease-free water). cDNA synthesis and target specific pre-amplification was done using Cell Direct one-step qRT-PCR kit (Life Technologies). Pre-amplification mastermix was added to each well; 6,25μL of 2X Reaction Buffer, 1μl of SuperScript III RT/Platinum Taq mix and 1.5μl of TaqMan assay mix. TaqMan assay mix was prepared by mixing equal volume of all target specific primers (*see* Table S1) (ThermoFisher). No-RT controls were prepared with Taq Polymerase (Invitrogen) and no SuperScript III RT enzyme was included. The PCR conditions were: 60 min at 50°C, 2 min at 95°C and 22 cycles (25 cycles for single cells) of 15 sec at 95°C and 4 min at 60°C. Pre-amplified product was diluted 1 to 5 and loaded onto a 48.48 chip together with Taqman universal MasterMix (Life Technologies) and the Taqman assays listed in Table S1 with the appropriate loading reagents according to manufacturer's instructions (BioMark 48.48 Dynamic array platform (Fluidigm)).

In some single cell sorts, index sorting was used and populations separated based on the index analysis. Single cell data are displayed as CT values (not normalized). Cells not expressing *GAPDH* were not considered further and in the analysis of ETV6-RUNX1 IPS cells, only cells expressing *ETV6-RUNX1* and *GAPDH* were considered. Co-expression of lineage-associated genes were counted according to the following rules: a cell is considered myeloid (GM) if expressing 2 of the myeloid genes, listed in red in Figure 3, lymphoid (L) if 2 of the genes listed in blue and B primed if expressing 2 of the genes listed in green respectively. Co-expression of GM and L and or B genes are labeled GM/L and GM/B respectively. Only cells expressing *IL7R* were considered in the count of the IL7R progenitor and only cells expressing *CD19* considered in the count of proB cells.

PCA analysis was performed on the single cell qPCR data. Genes for which the CT value was larger than 30 in all cells were discarded. All remaining CT values larger than 30 were truncated to 30. The scores were then inverted using the formula *30 –Ct*. TaqMan assays used in the PCA analysis are shown in the Table S1.

qPCR SYBR Green

hPSCs were lysed in Trizol (Invitrogen), RNA prepared by phenol extraction and isopropanol precipitation, treated with DNaseI (Promega) and cDNA produced from 1μg RNA by random primed SuperScript III (Invitrogen). Quantitative PCR was performed with SYBR Green (Applied Biosystems) using the primers for *OCT3/4* and *NANOG (see* Table S1*)* and normalized to *β-ACTIN*.

In Vitro Cultures

Stroma Co-cultures

To evaluate B lymphoid potential, indicated populations were plated on MS5 stroma. Media (a-MEM (Gibco), with 1% Penicillin/Streptomycin (HyClone), 1% L-glutamine (2mM, Gibco), 1% 2-Mercaptoethanol (0.1mM, Sigma-Aldrich) and 10% Fetal Bovine serum (FBS, HyClone) supplemented with cytokines) were changed weekly. Cytokines added are listed in Key Resources Table *(B)* (hFLT3L, hSCF, hTPO, hTSLP (only some experiments)).. After 2 weeks cells were picked and stained according to antibodies listed in Key Resources Table *B readout*. Clones were regarded as B cells if they had a $CD19^+$ phenotype.

Methocult

Indicated cell populations were plated in complete methylcellulose (MethoCult H4435, Stem CellTechnologies) and colonies scored at day 12-14. Some colonies could be picked and pooled and morphology evaluated on May-Grünwald (VWR International) and Giemsa (VWR International) stained cytospin slides.

Terasaki

Bulk sorted populations were plated as single cells or at a density of 20-40 cells/well in IMDM (HyClone) supplemented with 1% Penicillin/Streptomycin, 1% L-glutamine (2mM) and 20% FBS supplemented with cytokines according to Key Resources Table *Terasaki*. (hG-CSF, hIL-3, hFLT3L, hSCF, hTPO and in some experiments hM-CSF and hGM-CSF), Cultures were scored after 12-14 days of culture. Wells with cells were picked, pooled and morphology evaluated on May-Grünwald (Merck) and Giemsa (Merck) stained cytospin slides.

DJ Rearrangement

The IL7R progenitor (not purified on KIT) and control populations were sorted directly into KAPA Express DNA extraction Kit buffer (KAPABiosystems) and DNA extracted according to manufacture's recommendation. RT-PCR was performed mixing DNA with Maxima SYBR Green (ThermoFisher) and the primers listed in Table S1 (van Dongen et al., 2003). Two reactions were performed – one with D_H1-6 and the J_H consensus primers and one with D_H7 and the J_H consensus primer, the latter also rise gives to non-specific "germline" bands, but where positive produces bands in the region of 100-130bp. Samples were run with the following program: 15 min hold at 95°C followed by 50 cycles of 30 sec at 95°C, 30 sec at 60°C and 45 sec at 72°C and ending with one cycle of 15 sec at 95°C, 20 sec at 60°C and 15 sec at 95°C. The PCR product was run on a gel (2% Agarose, ethidium bromide) with TBE buffer (ThermoScientific). Genomic DNA from cells derived from myeloid culture was prepared either directly from Terasaki media into KAPA express DNA extraction buffer, or from stained cytospin slides by overnight demounting in xylene and direct washing in lysis buffer (NaCl 100mM, Tris-HCl pH 7.5 50mM, EDTA 10mM, SDS 0.5%, Proteinase K 0.8mg/ml). The gDNA was then subjected to a further purification step using 1:1 phenol:chloroform (Invitrogen), ethanol precipitated in the presence of 10μg glycogen carrier (Roche) and amplified as above.

Maintenance of hPSCs

MIFF3 IPS and H1 ES cells were cultured on matrigel-coated (Corning) 6 well plates in either mTeSR1 (Stem Cell Technologies) or StemFitAK (Ajinomoto Co., Inc, Tokyo, Japan) (See Key Resources Table). Cultures were passaged every 4-7 days using gentle dissociation reagent (SCT) according to manufacturer's instructions in the presence of Y27632 rock inhibitor (Miltenyi).

In Vitro B Cell Differentiation of hPSCs

In vitro B cell differentiation was performed as previously described (Carpenter et al., 2011; Vodyanik et al., 2005). OP9 stroma (kind gift from I Slukvin) were overgrown for 8-12 days on gelatinized 10cm plates in OP9 maintenance media (OP9-M: αMEM (Invitrogen), 20% batch tested defined FCS (Hyclone)**,** 100μM monothioglycerol (Sigma), 1x penicillin/streptomycin (Invitrogen)), with a 5ml half media exchange conducted on day 4. Large colonies of hPSCs were detached by incubation with 1mg/ml collagenase IV (Invitrogen) diluted in DMEM/F12 (Invitrogen) for 10-90 minutes. 1.5-2x10^6 hPSCs were seeded per 10cm dish in 10ml of OP9-D media (OP9-D: αMEM (Invitrogen), 10% defined FCS (Hyclone)**,** 100μM monothioglycerol, 1x penicillin/streptomycin). A complete media change was conducted on day 1, half media changes were conducted on days 4, 6 and 8 with $CD34^+$ cells harvested on day 10.

At day 10 media was removed and the OP9 matrix was digested with 7ml fresh collagenase IV 1mg/ml in DMEM/F12 at 37°C and 7ml fresh 0.05% trypsin (Invitrogen)/0.5mM EDTA (Sigma) at 37°C. After 7 minutes the reaction was quenched with 7ml OP9-D media. Once triturated the cells were strained through a 40μm nylon filter (BD Falcon). After centrifugation the cellular pellet was re-suspended and enriched using $CD34^+$ MACS beads (Miltenyi) according to manufacturer's instructions. Following enrichment the cells were re-suspended in 0.5-1ml OP9-D media, counted and seeded onto MS5 stroma.

MS5 were maintained in T75 flasks in MS5 maintenance media (MS5-M: αMEM, 10% batch-tested defined FCS (Hyclone)**,** 1x penicillin/streptomycin). 2x10^5 MS5 cells are seeded in MS5-M media onto 6 well plates 1-2 days prior to use for differentiation. 12.5-50x10^4 CD34 enriched cells were added to each well in 2ml OP9-D media supplemented with hIL3 10ng/ml, hIL7 20ng/ml, hFLT3L 50ng/ml, hSCF 50ng/ml (See Key Resources Table). 2ml of the above media (excluding IL3) was added at day 7. A further

media change is performed at day 14. Cells are harvested after 21 days of MS5 co-culture. For the competitive co-culture equal numbers of wild type MIFF3 and ETV6-RUNX1 $CD34^+$ cells from day 10 of OP9 co-culture (determined via manual counting after magnetic bead enrichment or by FACS sorting) were seeded together onto MS5 stroma. Cells were cultured as per the B cell differentiation protocol and analyzed at day 29-31.

Genome Engineering

A homologous recombination vector targeting *ETV6* intron V was constructed by BAC recombineering (RP11_418C2 Source Biosciences). BAC-containing DH10B E. coli were grown overnight in 1.4ml of LB/chloramphenicol (10μg/ml). On day 2 an outgrowth of this culture was made recombineering proficient by electroporation with the pSC101-BAD-gbaA-tet plasmid (recombineering plasmids provided by Francis Stewart, University of Dresden). Cells were recovered at 30°C in 1ml SOC for 2 hours without selection and after recovery 100μl of the culture was inoculated into a new tube containing 900μl LB chloramphenicol (10 μg/ml) and tetracycline (4 μg/ml) and incubated at 30°C for 20 hours.

The homology arms for the ETV6 targeting vector were subcloned from the BAC by gap-repair. *Xma*I linearized p15a-DTA-AmpR backbone plasmid was amplified by Phusion high-fidelity PCR (NEB) using PAGE-purified primers designed with 50bp homology arms aligned for gap-repair (Primers; See Table S1) followed by *Dpn*I digestion of plasmid template. Following arabinose induction the E. coli were electroporated with 400ng of cleaned ETV6-specific p15a-DTA-AmpR PCR product. DNA and arabinose negative controls were also included. The cells were recovered in SOC for 2 hours at 30°C then overnight cultures in 1.4ml LB tetracycline (4μg/ml) and ampicillin (50μg/ml) at 30°C. The following day the E. coli were arabinose induced and electroporated with 200ng of a column-purified PCR product of the rpsL-GentaR positive/negative selection cassette containing 50bp homology arms targeting the cleavage point of ETV6 CRISPR gRNA (Primers; See Table S1). The cells were recovered in SOC for 2 hours at 30°C then cultured overnight in 1.4ml LB with tetracycline (4μg/ml), ampicillin (50μg/ml) and gentamicin (4μg/ml).

The following day the rpsL/GentaR positive negative selection cassette was replaced with the RUNX1 knock in cassette. The cassette was designed *in silico* and commercially synthesized as in two fragments (GeneArt). These were released by *Xho*I *Sbf*I double digest and ligated into a pR6K-AmpR plasmid backbone in $pir116^+$ E coli to prevent background transformation during recombineering. The knock in cassette flanked by 50bp homology arms targeting the knock in site was released from a pR6K plasmid backbone by *Asc*I *Not*I double digest. Following arabinose induction the E. coli were electroporated with 600ng of linearized knock in construct. DNA and arabinose negative controls were also included. The cells were recovered in SOC for 1 hour at 37°C then cultured overnight in 1.4ml LB with ampicillin (50μg/ml) and kanamycin (5μg/ml) at 37°C. Plasmid DNA was extracted from the cultures and retransformed into chemically competent E. coli, plated on Kan/Amp agar and clones verified by restriction digest and sequencing across all recombineering junctions and the knock in cassette.

Nucleofection and Selection of hPSCs

MIFF3 hPSCs were harvested as single cells using gentle dissociation reagent following 2 hours pre-incubation with rock inhibitor. $2x10^6$ hPSCs were transfected with 2ug *Xho*I linearized RUNX1 knock-in construct and 4ug each of Cas9*D10A* (a gift from George Church (Addgene plasmid #41816), modified to include a 5'NLS, kindly provided by Dr Elspeth Payne, UCL Cancer Institute) and a single custom *ETV6* guide RNA (pBS-ETV6gRNA: target GGCAATTGGAGGCTTCTGCT) using human stem cell nucleofector kit 2 using programme B016, Nucleofector IIA (Lonza). Cells were recovered into 10ml mTeSR1/Y27632 on a 10cm matrigel-coated plate. Media was changed daily and at 48 hours 50μg/ml G418 selection was commenced. At day 8-10 single colonies were picked under microscopic guidance into a 96 well plate. When confluent clones were replica plated: one plate was frozen at -80°C in 90% knockout serum replacement (Invitrogen)/10% DMSO (Sigma) supplemented with Y27632; the other was lysed for screening by southern blot at 65°C overnight in 50μl cell lysis buffer (NaCl 100mM, Tris-HCl pH 7.5 50mM, EDTA 10mM, SDS 0.5%, Proteinase K 0.8mg/ml).

Screening by Southern Blot Hybridization

96 well plates of cell lysates underwent DNA precipitation by the addition of 50μl isopropanol. The plate was vortexed gently and incubated at room temperature for 30mins and DNA pelleted by centrifugation at 2,000g for 20mins. After 3 washes in 70% ethanol and drying for 20mins at room temperature 30μl KpnI-HF digest mix was added (1x NEB CutSmart® buffer, 0.2mg/ml BSA, 1mM spermidine, 20μg/ml RNaseA, 10U/30μl KpnI-HF) and the DNA digested overnight at 37°C. The following morning 5μl KpnI-HF digest mix was added (10U/5μl). After 4 hours of further digestion 3μl of 10x loading buffer is added and 2x 96 well plates run on a 200well 0.8% agarose gel in TAE at 100V for 2-3 hours alongside a λ*Hind*III ladder (NEB). The gel was briefly photographed in a UV transilluminator. The gel was depurinated in 250mM HCl for 10mins, denatured in 2x 15min 0.5M NaOH/1.5M NaCl, neutralized in 1.5M NaCl/TrisHCl 0.5M pH7.5 and equilibrated in 20xSSC for 10mins. The DNA was transferred overnight by capillary transfer onto a positively charged nylon membrane (Roche). The following morning the nylon membrane was UV cross-linked (120mJ Stratalinker) and the gel stained with ethidium bromide and visualized to confirm complete transfer of DNA.

The nylon membrane was pre-hybridised at 42°C in DIG easyhyb buffer for 60mins (Roche). An external Southern probe (SP2) was produced using HotStar® Taq polymerase (Qiagen) from 500pg ETV6 BAC template (Primers see Table S1). Conditions: 95°C 15min, 94°C 30s, 56°C 30s, 72°C 2min, repeat 35 cycles, 72°C 10min. 100ng of cleaned SP2 PCR product was labeled with ^{32}P-dATP using random primed Klenow fragment (Agilent). A λ*Hind*III probe was labeled in the same way and the labeled DNA was column purified (GE). The probes were melted for 5min at 95°C before hybridizing overnight at 42°C. The next day the membrane was washed twice in

low stringency buffer (0.5xSSC/0.1%SDS) for 5min and twice in high stringency buffer (0.1XSSC/0.1%SDS) at 68°C for 15min. The membrane was then sealed and exposed to a phosphor screen for 24-48 hours.

Knockin hPSC Colony Expansion and Checking

Candidate knock in clones were identified from screening Southern blot and thawed onto HS27 human feeder stroma grown on a matrigel coated 24 well plate in mTeSR1 supplemented with Y27632. hPSC colonies were passaged onto matrigel coated 6 well plates, stocks frozen and DNA prepared for confirmatory southern blot including internal *Neo* probing of *Bam*HI digested genomic DNA (Primers see Table S1).

Immunostaining

hPSC colonies were fixed in 4% paraformaldehyde and permeabilized for 2 hours (PBS w/o Ca/Mg, 1% BSA, 3%FCS, 0.1% TritonX100) before primary staining overnight with OCT3/4 (SantaCruz 1:200; Secondary stain Cy5 anti-Rabbit IgG 1:1000 Jackson Labs).

Karyotyping

Confirmation of normal karyotype by G banding was kindly performed by Duncan Baker (Sheffield Diagnostic Genetics Service) and Dr Christian Unger (Centre for Stem Cell Biology, University of Sheffield).

Western Blot

Sorted $CD45^{+}Venus^{+}$ hIPSC-derived cells or cultured REH (cell line carrying ETV6-RUNX1 fusion) were washed in PBS, pelleted, re-suspended in cold RIPA buffer (50mM Tris pH8.0, 150mM NaCl, 1% Igepal CA-630, 0.5% Na-deoxycholate, 0.1% SDS) containing protease inhibitors (cOmplete Mini Protease Inhibitor Cocktail, Roche 11836153001) and incubated on ice for 10 minutes. Lysates were centrifuged at 20,000g for 10 minutes to pellet the insoluble fraction, supernatants were removed and pellets washed 1x in RIPA. Pellets were resuspended in RIPA and sonicated in a Bioruptor Pico (Diagenode) for 5 x 30seconds. Samples were subjected to PAGE and western blotting. ETV6-RUNX1 was detected with an antibody against RUNX1 (Abcam, ab23980, 1ug/ml). Histone H3 was used as a loading control (Abcam ab1791, 1ug/ml). Cell lines NALM6 (N6) and MIFF3 were used as negative controls.

Removal of the RUNX1 Cassette by Cre Recombinase

10 000 hPSCs from *ETV6-RUNX1* knock in clone #2.8 were seeded into a matrigel coated 12 well plate in StemFitAK media. The following day the cells were incubated for 3 hours in 500μl StemFitAK media containing 5μM TAT-Cre (Millipore). hPSCs were passaged after 48 hours as single cells and plated at limiting dilution. Emerging colonies were picked under microscopic guidance into 96 well plates and processed as above for screening by Southern blot hybridization (*Kpn*I-HF digest, *Neo* probe). By designing the reverted clones to continue to harbor the NeoR selection cassette provided a KI specific Southern probe that can be used to screen for Cre reverted cells, giving us confidence that these control lines are not sub-clones of contaminating wild type cells. Furthermore, the ongoing presence of the NeoR cassette allows us to maintain the knock in hPSCs and their reverted clones in G418 selection, reducing the risk of background contamination by WT hPSCs.

RNA-Seq

22-400 cells were directly sorted into 800μl Trizol and frozen at -80°C. RNA was extracted in the aqueous phase after addition of 160ul chloroform, precipitated with an equal volume of isopropanol supplemented with 20μg linear polyacrylamide and washed once in fresh 80% ethanol. The RNA pellet was re-suspended in 3μl water at 50°C for 10 minutes and then kept on ice. 0.5-1μl of RNA was quantitated using an Agilent Bioanalyser RNA 6,000 pico chip. 100pg (or 1μl of stock if unquantifiable) of RNA used for first strand synthesis using SMARTER v3 (Clontech) before 16 cycles of amplification according to manufacturer's instructions. cDNA was purified on Agencourt AMPureXP magnetic beads, washed twice with fresh 80% ethanol and eluted in 17μl elution buffer. 1μl cDNA was checked and quantified on an Agilent Bioanalyser high sensitivity DNA chip. Sequencing libraries were produced using Illumina Nextera XT tagmentation according to manufacturer's instructions except using 150pg input cDNA, 5mins tagmentation and amplification 12 cycles using Illumina XT 24 index primer kit. Libraries were cleaned using an equal volume (50μl) Agencourt AMPureXP magnetic beads and re-suspended in 20μl elution buffer. Libraries were checked and quantified on an Agilent Bioanalyser high sensitivity DNA chip (size range 150-2000bp) and by Qubit dsDNA BR (Molecular Probes). Libraries were pooled to a normalized concentration of 1.5nM and sequenced on an Illumina® NextSeq 500 using 150bp paired end kit as per manufacturer's instructions. See also Key Resources Table.

Bioinformatics

Raw reads were initially processed with PRINSEQ-lite v0.20.4 (Schmieder and Edwards, 2011) to trim low-quality reads. They were further aligned with TopHat2 v2.0.11 (Kim et al., 2013) on the GRCh37 human assembly and FPKM values for protein-coding genes were calculated with Cufflinks v2.2.0 (Trapnell et al., 2010) using uniquely mapping reads only. Genes with an FPKM < 5 in every sample were filtered out. The remaining ones were log-transformed using the formula log2 (FPKM+1). Finally, expression values were normalised upon quantiles (Bolstad et al., 2003) prior to the PCA analysis. When building the PCA map based on the expression in primary samples only (FL CS17, FL CS21-22 and Adult BM), the FL CS17 $IL7R^{+}$ Progenitor sample was detected as a clear outlier.

A new map based on all other primary cells was used instead and the FL CS17 IL7R+ Progenitor sample was projected on the new map. When building the PCA map based on the expression of all wild-type samples (hPSC D31 and the aforementioned primary samples), the FL CS17 IL7R+ Progenitor sample rested on the correct region of the PCA map (Figure S3E). Gene Set Enrichment Analysis (GSEA) was performed with permutation type=gene set and the gene sets used were obtained from Laurenti et al. (Laurenti et al., 2013; Mootha et al., 2003; Subramanian et al., 2005). Ellipses on the PCAs were calculated using ggplot stat_ellipse (http://ggplot2.tidyverse.org/reference/stat_ellipse.html) assuming a multivariate t-distribution and a confidence level of 0.9.

QUANTIFICATION AND STATISTICAL ANALYSIS

Data are shown as mean values with standard deviation (SD) if not stated otherwise. Statistics was performed using a non-parametric Mann-Whitney test or Kruskal-Wallis/Dunns test for multiple comparisons. In Figure 2B, CB was used as control group for the statistical comparisons made and in Figure 5C MIFF3 was used as control group.

DATA AND SOFTWARE AVAILABILITY

The accession number for the RNASeq data reported in this paper is ArrayExpress: E-MTAB-6382.

Developmental Cell, Volume *44*

Supplemental Information

A Human IPS Model Implicates Embryonic B-Myeloid Fate Restriction as Developmental Susceptibility to B Acute Lymphoblastic Leukemia-Associated ETV6-RUNX1

Charlotta Böiers, Simon E. Richardson, Emma Laycock, Alya Zriwil, Virginia A. Turati, John Brown, Jason P. Wray, Dapeng Wang, Chela James, Javier Herrero, Ewa Sitnicka, Stefan Karlsson, Andrew J.H. Smith, Sten Erik W. Jacobsen, and Tariq Enver

SUPPLEMENTAL INFORMATION

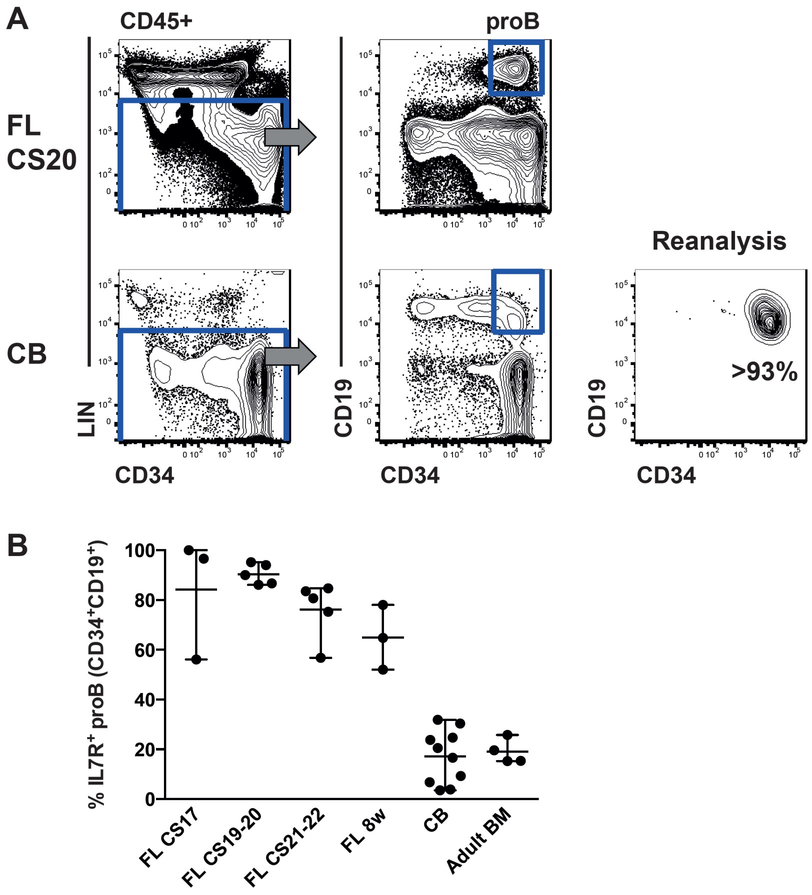

Figure S1 (related to Figure 1)

A. Flow cytometry profile showing gating strategy of proB cells ($CD34^{+}CD19^{+}$). Cells were first gated for viability, singlets and scatter and after that for CD45 positivity. Further gating is indicated in the figure. Reanalysis was normally done on cord blood, due to the low cell number that could be obtained from the fetal samples. ProB cells were typically sorted with >93% purity as indicated in the right panel.
B. Percentage proB cells expressing surface IL7R at different time points of development. Each dot represents one biological sample. Mean with range.

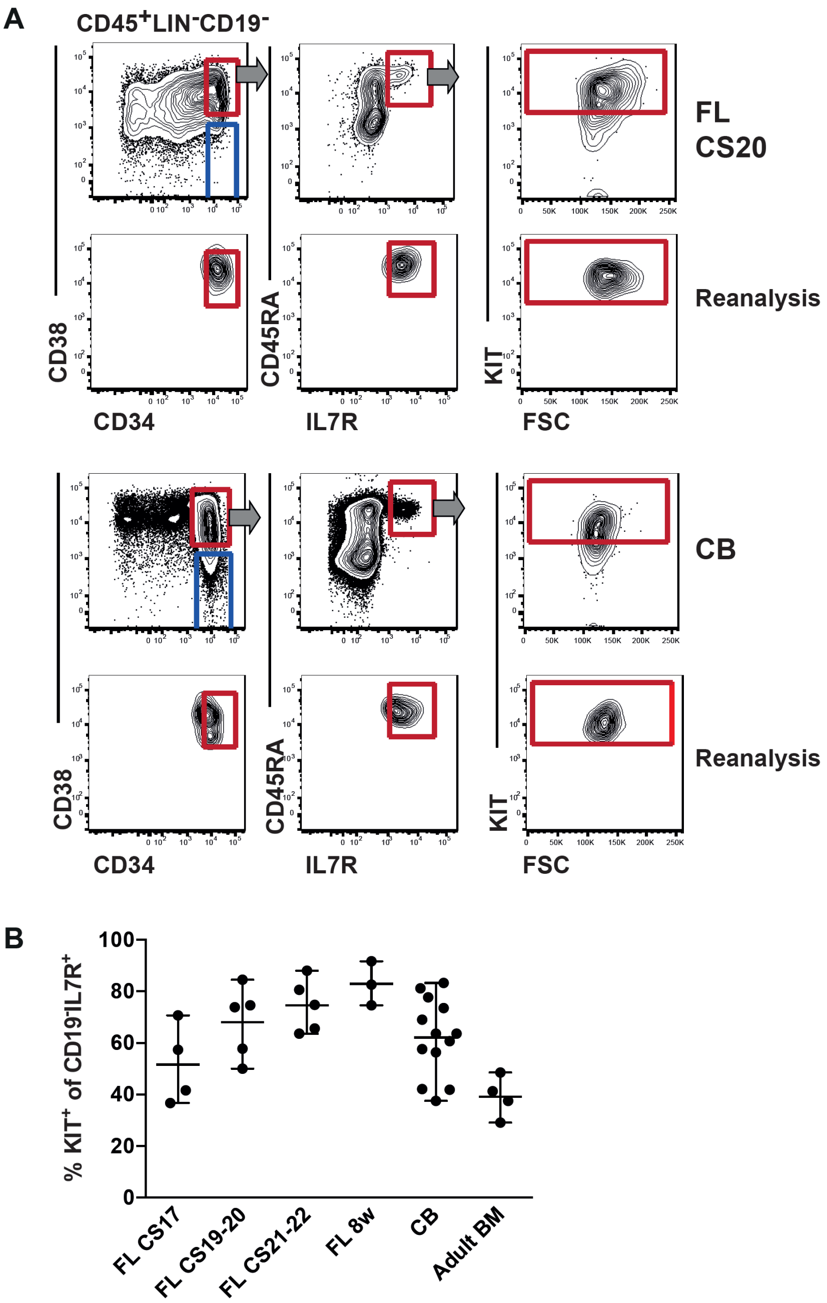

Figure S2 (related to Figure 2)

A. Flow cytometry profiles showing sorting strategy and reanalyses for $IL7R^{+}KIT^{+}$ progenitors. Viable cells were gated for CD45 positivity, then LIN and CD19 negativity. Further gating as indicated in the figure. $CD34^{+}CD38^{-}$ gate is indicated in blue. Reanalyses are shown for FL (top) as well as CB (bottom), but were normally done on CB and only occasionally on FL, due to the low cell number that could be obtained from the fetal samples. The $IL7R^{+}KIT^{+}$ progenitor was typically sorted with >94% purity.
B. Percentage of $IL7R^{+}$ progenitor cells expressing KIT at different time points of development. Each dot represents one biological sample. Mean with range.

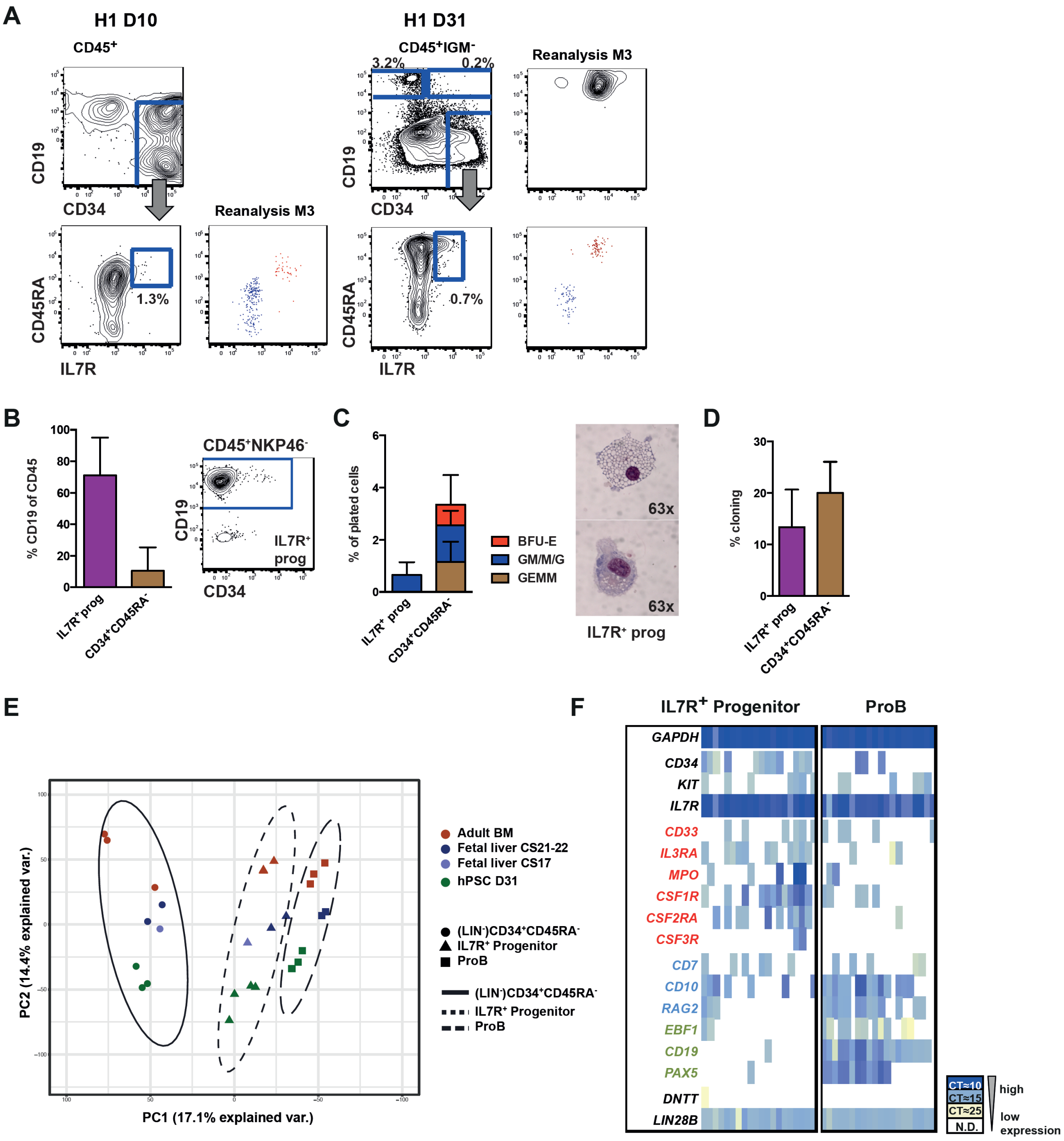

Figure S3 (related to Figure 4)

A. H1 hESCs were analyzed for CD19^{+} B cells (**top**) and IL7R^{+} progenitor (**bottom**) at D10 (**left**) and D31 (**right**) of differentiation. Cells were gated CD45^{+} (D10) or CD45^{+}IGM^{-} (D31). Further gating is indicated in the figure. Numbers show mean percentages of CD45^{+} cells. Representative reanalysis of MIFF3 hPSCs is shown to the right of each plot, IL7R^{+} progenitor (red) and CD34^{+}CD45RA^{-} control cells (blue), proB cells right top panel. Populations were typically sorted with ≥94% purity.

B. B cell potential of the MIFF3 derived IL7R^{+} progenitor (D31) was investigated on MS5 stroma. CD34^{+}CD45RA^{-} cells were cultured in parallel. Graph shows percent CD19^{+} cells of total CD45^{+} cells in the culture. Mean+SD, n=2. To the right: representative FACS analysis of the IL7R^{+} progenitor readout.

C. Myeloid potential of the IL7R$^+$ progenitor (derived from MIFF3 and H1 at D31) was investigated in semi-solid media. Primitive CD34$^+$CD45RA$^-$ cells (also derived from hPSCs) were cultured in parallel. Graph shows percent colonies per plated number of cells. Mean+SD, n=2. To the right: typical macrophages derived from IL7R$^+$ progenitor culture.

D. Myeloid potential of single hPSC-derived IL7R$^+$ progenitors (MIFF3 at D24-27) was investigated in liquid culture. Graph shows percent of wells with >10 cells. Primitive CD34$^+$CD45RA$^-$ cells were cultured in parallel. Mean+SD, n=3.

E. PCA calculated using RNA sequencing data from both human primary samples and hPSCs from D31 ((LIN$^-$)CD34$^+$CD45RA$^-$, IL7R$^+$ progenitor and proB cells) of differentiation. Note that the IL7R progenitor compartment from very early CS17 FL here rests on the correct region of the PCA map whereas it is an outlier on the PCA built on only primary samples (Figure 4D). PC1 and PC2 are shown and each dot represents one individual sample. The ellipses show the 90% density function for that cell type. Legend is shown to the right.

F. Single cell qPCR analysis of IL7R$^+$ progenitor and proB cells from H1 hESCs at D31 of differentiation. Each column represents one single cell. Only cells expressing *GAPDH* are shown. Color code based on CT values. Genes labeled in red are myeloid, blue lymphoid and green B cell genes, n=1, 20 cells investigated/population.

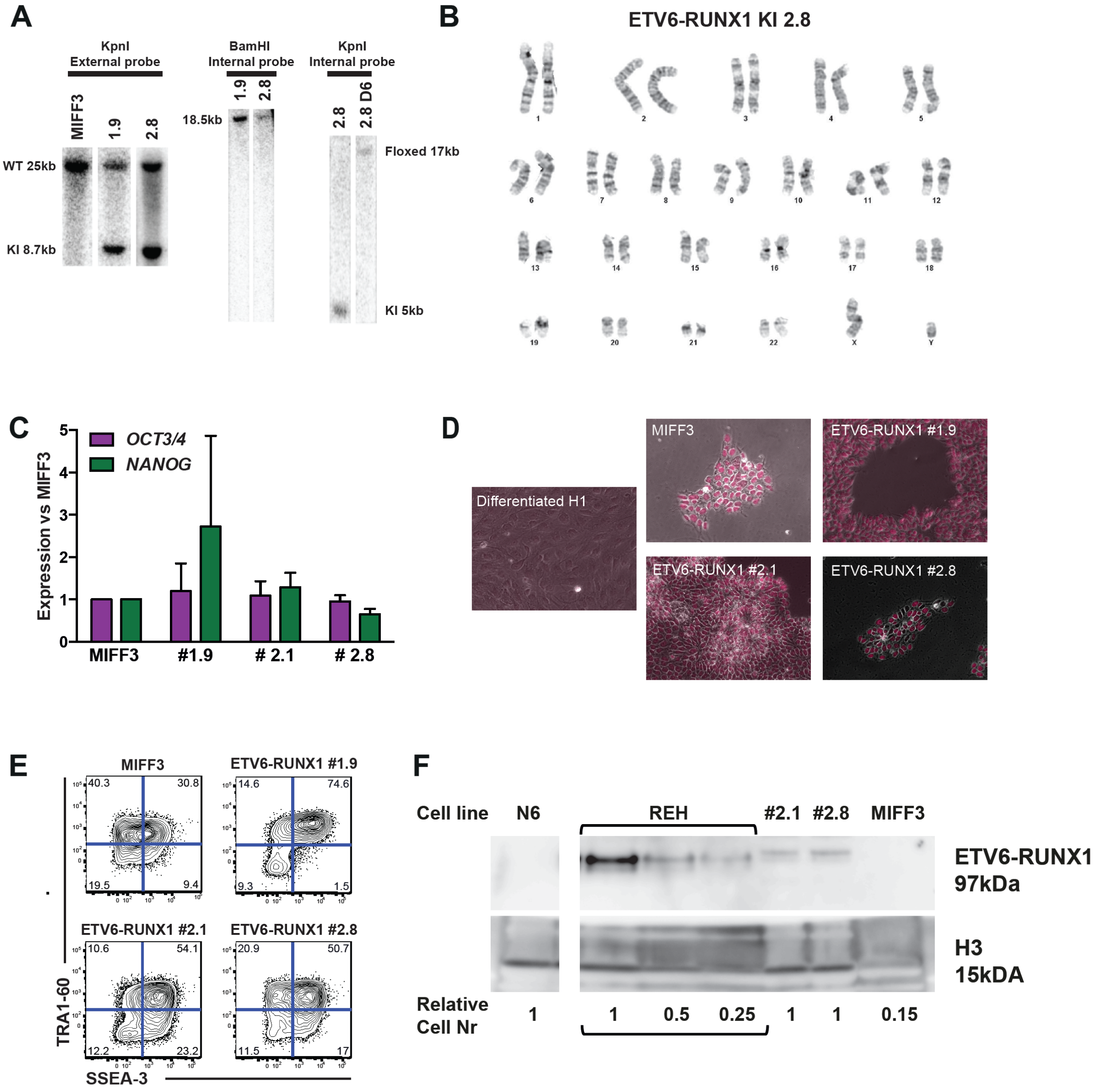

Figure S4 (related to Figure 5)

A. Representative southern blot hybridizations of genome engineered MIFF3 hIPSCs. Left: Successful targeting of *ETV6* allele as shown by external *ETV6* probe on *Kpn*I digested DNA (WT 25kb, KI 8.7kb). Middle: Internal probe (*neoR*) on *Bam*HI digested DNA showing single insertion at 18.5kb. Right: Knock in clone #2.8 before (left - KI 5kb) and after (right - reverted 17kb) floxing with cre recombinase using internal (*neoR*) probe on *KpnI* digested DNA.
B. Karyotype of middle passage ETV6-RUNX1 knock-in hPSC line #2.8.
C. Quantitative gene-expression analysis of unmodified MIFF3 and 3 successfully targeted ETV6-RUNX1 knock in clones for the pluripotency genes *OCT3/4* and *NANOG*. Expression is normalized to *β ACTIN* and presented relative to MIFF3. Mean+SD, n=2.
D. Immunostaining for nuclear OCT4 on fixed MIFF3 and 3 different ETV6-RUNX1 knock-in clones. Differentiated H1 is shown as control.

E. Flow cytometry analysis of the pluripotency surface markers TRA1-60 and SSEA3 in MIFF3 and 3 different ETV6-RUNX1 knock-in clones.
F. Western blot of ETV6-RUNX1 protein from FACS sorted hematopoietic progenitors ($CD45^{+}Venus^{+}$) derived from ETV6-RUNX1 knock in #2.1 and #2.8. Positive control: ETV6-RUNX1 B-ALL cell line (REH). Negative controls: MIFF3 and NALM6 (N6). Note knock-in ETV6-RUNX1 protein includes additional V5 tag. One lane has been removed but all samples were run on the same gel.

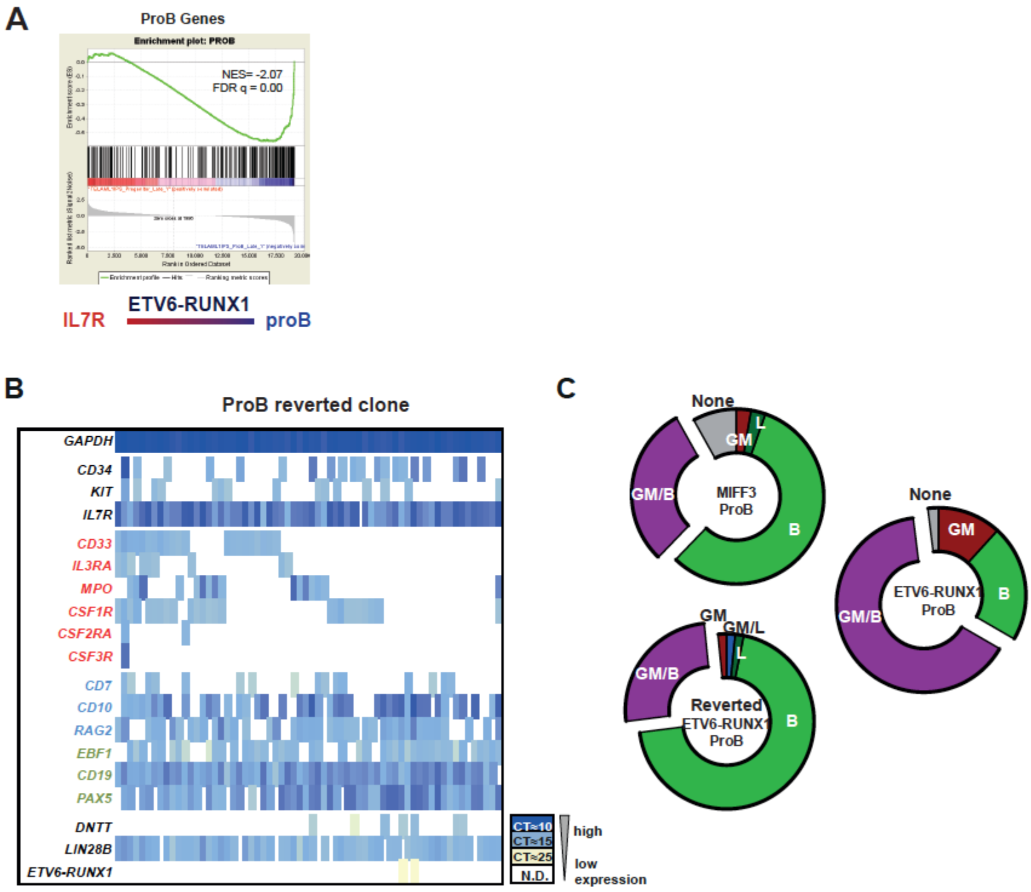

Figure S5 (related to Figure 6)

A. Gene set enrichment analysis (GSEA) comparing the proB cell gene expression of ETV6-RUNX1^{+} IL7R progenitor and ETV6-RUNX1^{+}proB cells.
B. Single cell qPCR analysis of proB cells (CD34^{+}CD19^{+}) from ETV6-RUNX1 reverted clone. Each column represents one single cell. Only cells expressing *GAPDH* are shown. Color code according to CT values. Gene sets: red, myeloid; blue, lymphoid; green, B lineage related. n=2, 64 cells investigated.
C. Co-expression of lineage associated genes based on single cell data in Figure 6E, and Figure S5B. A cell is considered myeloid (GM) if expressing 2 of the myeloid genes, lymphoid (L) if 2 of the genes listed in blue and B primed (B) if expressing 2 of the genes listed in green. Co-expression of GM with L or B genes are labeled GM/L and GM/B respectively (see *STAR Method*). Data from D31 proB are shown (MIFF3, ETV6-RUNX1 hIPS and ETV6-RUNX1 hIPS reverted clones).

Table S1: **(related to STAR Methods)**

Oligonucleotides

Oligonucleotides	Source	Identifier	Used for PCA
CCR9	ThermoFisher	Hs00258535_m1	x
CD10	ThermoFisher	Hs00153510_m1	x
CD19	ThermoFisher	Hs00174333_m1	x
CD33	ThermoFisher	Hs01076281_m1	x
CD34	ThermoFisher	Hs00990732_m1	x
CD38	ThermoFisher	Hs01120071_m1	x
CD62L (SELL)	ThermoFisher	Hs00174151_m1	x
CD7	ThermoFisher	Hs00196191_m1	x
CKIT	ThermoFisher	Hs00174029_m1	x
CRLF2	ThermoFisher	Hs00845692_m1	x
CSF1R (M-CSFR)	ThermoFisher	Hs00911250_m1	x
CSF2RA (GM-CSFR)	ThermoFisher	Hs00538900_m1	x
CSF3R (G-CSF R)	ThermoFisher	Hs01114427_m1	x
DNTT	ThermoFisher	Hs00172743_m1	x
E2A (TCF-3)	ThermoFisher	Hs00413032_m1	x
EBF1	ThermoFisher	Hs00395513_m1	x
EPOR	ThermoFisher	Hs00181092_m1	x
ETV6	ThermoFisher	HS00231101_m1	
*ETV6-RUNX1**	ThermoFisher	F *TEL*: CTCTGTCTCCCCGCCTGAA<br>P *TEL*; TCCCAATGGGCATGGCGTGC<br>R *AML1*; CGGCTCGTGCTGGCA	
FLT3	ThermoFisher	Hs00174690_m1	x
GATA1	ThermoFisher	Hs01085823_m1	x
GATA3	ThermoFisher	Hs00231122_m1	x
GAPDH	ThermoFisher	Hs99999905_m1	x
IGHM	ThermoFisher	Hs00378435_m1	x
IKAROS	ThermoFisher	Hs00172991_m1	x
IL2RG	ThermoFisher	Hs00953624_m1	x
IL3RA	ThermoFisher	Hs00608141_m1	x
IL7R	ThermoFisher	Hs00902334_m1	x
LIN28B	ThermoFisher	Hs01013729_m1	x
MPL	ThermoFisher	Hs00180489_m1	x
MPO	ThermoFisher	Hs00165162_m1	x
NOTCH1	ThermoFisher	Hs01062011_m1	x
PAX5	ThermoFisher	Hs00277134_m1	x
PRETa	ThermoFisher	Hs00300125_m1	x
PU.1	ThermoFisher	Hs02786711_m1	x
RAG2	ThermoFisher	Hs00379177_m1	x
TCF7	ThermoFisher	Hs00175273_m1	x
VWF	ThermoFisher	Hs00169795_m1	x
OCT3/4	IDT	F: GAAGGAGAAGCTGGAGCAAA	N/A

		R: CTTCTGCTTCAGGAGCTTG	N/A
NANOG	IDT	F: GATTTGTGGGCCTGAAGAAA	N/A
		R: CAGGGCTGTCCTGAATAAGC	N/A
β-ACTIN	ThermoFisher	Hs99999903_m1	N/A
D_H1	IDT/Sigma	GGCGGAATGTGTGCAGGC	N/A
D_H2	IDT/Sigma	GCACTGGGCTCAGAGTCCTCT	N/A
D_H3	IDT/Sigma	GTGGCCCTGGGAATATAAAA	N/A
D_H4	IDT/Sigma	AGATCCCCAGGACGCAGCA	N/A
D_H5	IDT/Sigma	CAGGGGGACACTGTGCATGT	N/A
D_H6	IDT/Sigma	TGACCCCAGCAAGGGAAGG	N/A
D_H7	IDT/Sigma	CACAGGCCCCCTACCAGC	N/A
JH consensus	IDT/Sigma	CTTACCTGAGGAGACGGTGACC	N/A
5' gap-repair	IDT	GCTAATATATATAATATATTATATATACGAATATACTAATTATGGCTGACTTAATA AGATGATCTTCTTGAGATCG	
3' gap-repair	IDT	CGTAAAAGTTCTGTCATTAACTGAATAGCCATAAAAAGCCTTTAAATCTCTTACC AATGCTTAATCAGTGAGG	
5' rpsL-genta	IDT	CACCATTCCCAATTAGGCGCCCTCCAAGGCTCTCTGAGGGCAATTGGAGGCATTA ATTCCTAATTTTTGTTGAC	
3' rpsL-genta	IDT	AGCGGCTCATTAACCAAGCCATTAGGGATTTAGCCTCATCCAAGCAGAAGTTAGG TGGCGGTACTTGGG	
ETV6 Southern probe F	IDT	CCCATCTGAGAGGGACTGTG	N/A
ETV6 Southern probe R	IDT	GTATCCGCAGCTAAAGGCATTG	N/A
neoR Southern probe F	IDT	GCTATTCGGCTATGACTGGG	N/A
neoR Southern probe R	IDT	GAAGGCGATAGAAGGCGATG	N/A

* Gabert et al., *Leukemia*, 2003
